# Ultrasmall radical metal organic cage as cascade antioxidant nanozyme for renal injury

**DOI:** 10.7150/thno.105807

**Published:** 2025-01-27

**Authors:** Cheng Huang, Ziyu Liu, Yucen Deng, Xiaoyan Wang, Qing Miao, Demei Sun, Xinyuan Zhu, Jinghui Yang, Youfu Wang

**Affiliations:** 1 School of Chemistry and Chemical Engineering, Shanghai Jiao Tong University, Shanghai 200240, China.; 2 Department of Nephrology, Shanghai Changzheng Hospital, Naval Medical University, Shanghai 200003, China.; 3 School of Pharmacy, China Medical University, Shenyang 110122, China.; 4 Shanghai Center for Systems Biomedicine, Shanghai Jiao Tong University, Shanghai 200240, China.; 5 Department of Organ Transplantation, Shanghai Changzheng Hospital, Naval Medical University, Shanghai 200003, China.

**Keywords:** metal organic cage, cascade nanozyme, antioxidant, ischemia reperfusion injury, kidney.

## Abstract

**Rationale:** As substitutes for natural enzymes, nanozymes offer tunable enzyme-like activities and remarkable structural stability, granting them the potential to treat various diseases, including renal ischemia-reperfusion (I/R) injury. However, the majority of developed nanozymes suffer from unclear structures and limited activity profiles, which hinder the study of their structure-activity relationships, catalytic diversity, mass production, and clinical application.

**Methods:** Herein, we introduce an atomically precise and ultrasmall cascade nanozyme based on a radical-functionalized metal-organic cage (MOC-R). This nanozyme is synthesized through the coordination of radical ligands with copper ions, resulting in a cuboctahedral structure.

**Results:** The MOC-R exhibits cascade antioxidant activities, mimicking the functions of superoxide dismutase (SOD) and catalase (CAT), owing to the synergism between the external radicals and internal copper clusters. The MOC-R nanozyme demonstrates exceptional radical scavenging and anti-inflammatory properties. It mitigates immune cell infiltration, promotes macrophage polarization towards the M2-like phenotype, reduces inflammatory cytokine secretion, and suppresses excessive autophagy and apoptosis.

**Conclusions:** This study not only presents an atomically precise cascade nanozyme but also highlights its promising therapeutic potential for renal I/R injury.

## Introduction

Clinically, renal I/R injury is a significant concern in various medical and surgical settings. This condition commonly occurs during procedures such as partial nephrectomy, kidney transplantation, and aortic surgery 1. In these scenarios, the temporary cessation of blood flow to the kidneys followed by its re-establishment can lead to severe damage, causing acute tubular necrosis. The underlying mechanisms responsible for renal I/R injury are complex and multifaceted, involving several interconnected pathways. One of the key factors is the excessive production of intracellular reactive oxygen species (ROS). Under normal conditions, ROS are produced in small amounts and play essential roles in cellular signaling and homeostasis. However, during ischemia, the lack of oxygen leads to mitochondrial dysfunction, and upon reperfusion, there is a sudden influx of oxygen that triggers an overproduction of ROS. Given the central role of ROS in this process, the administration of antioxidants has been explored as a potential therapeutic strategy. Various studies have demonstrated promising results in experimental models, where antioxidants effectively mitigated tissue damage and improved renal function. However, translating these findings into clinical practice has proven challenging. Drugs designed to scavenge ROS generated during reperfusion often face limitations in efficacy due to their unsustainable one-time-exhausted character and poor accumulation at the injured site 2,3. Enzymes, as remarkable biological catalysts, play a crucial role in facilitating chemical reactions within living organisms. One of the key characteristics of enzymes is that they remain structurally and chemically unchanged before and after the reactions they catalyze. This unique property allows enzymes to be reused multiple times without losing their functionality 4-8.

However, natural enzymes possess a level of complexity and instability that complicates their synthesis, large-scale production, and long-term preservation. The intricate three-dimensional structures of natural enzymes are highly sensitive to environmental factors such as temperature, pH levels, and organic solvents. Even minor changes in these conditions can lead to denaturation, where the enzyme loses its functional shape and, consequently, its catalytic ability. This sensitivity poses significant challenges for industrial and clinical applications, particularly when it comes to maintaining enzyme activity over extended periods or under varying conditions. Moreover, the difficulty in producing natural enzymes on a large scale and preserving them effectively has hindered their widespread use in clinical settings. In medical treatments, enzymes are often required to remain stable and active for prolonged durations, which is not always feasible with natural enzymes due to their inherent instability. This limitation has spurred the development of artificial enzymes designed to mimic the functionality of natural enzymes while offering enhanced structural stability 9,10. Nanozymes, emerging from advancements in nanoscience, represent a promising class of artificial enzymes 11,12. They offer benefits such as structural stability, multifunctionality, low cost, recyclability, and feasibility for large-scale production 13,14. After nearly a decade of evolution, various nanozymes with diverse structures have been developed to alleviate various diseases and manifested outstanding physiological activities 15-18. Despite their potential, several challenges persist: 1) The often ambiguous structures of nanozymes complicate the study of their structure-activity relationships and their controlled large-scale preparation, which hinders clinical application; 2) The lack of standardized protocols for evaluating catalytic activity makes it difficult to compare different nanozymes; 3) Many nanozymes exhibit limited and monotonic activity, which restricts their therapeutic efficacy; 4) The biological safety of nanozymes remains uncertain, posing risks to clinical use 4,7,19-21. Addressing these issues by developing nanozymes with well-defined structures, versatile enzyme-like activities, and superior biocompatibility is critical for advancing their practical applications.

To address the challenges associated with nanozymes, we employed a well-defined nanoobject integrating various active components. The metal-organic cage (MOC) offers an atomically precise structure, presenting a uniform, discrete, and ultrasmall nanopolyhedron formed through the coordination of metal ions or clusters with organic ligands 22-26. MOCs integrate the advantages of both organic and inorganic components, offering several key benefits: 1) By modifying the organic linkers and metal nodes, researchers can tailor MOCs to possess varying nanoscale cavities and dimensions. This tunability allows for precise control over pore size and surface area, enhancing their functionality in applications such as catalysis and drug delivery. 2) Direct coordination of metal ions can shorten the spatial distance between catalytic sites, facilitating improved cascade catalysis. This arrangement promotes efficient multi-step reactions by ensuring that intermediates are readily accessible to subsequent active sites. 3) MOCs exhibit a unique cavity structure that enables reaction molecules to easily enter the cavities and interact with catalytically active sites from both inside and outside. This accessibility enhances catalytic efficiency and selectivity 27-29. Due to these structural features, functionality, and catalytic activity of metal sites, MOCs have found extensive use in the biomedical field 30,31. Actually, we have successfully constructed MOC-based cascade nanozymes recently, such as heterometallic MOC and oxidized MOC, demonstrating excellent enzymatic activity 32-34. However, despite these advantages, the atomic-level structure of these MOCs remains unclear, complicating detailed structural analysis and understanding of their active sites. Additionally, while these MOCs exhibit adequate biocompatibility, concerns remain regarding potential metallic toxicity 35-37.

**Scheme 1:**
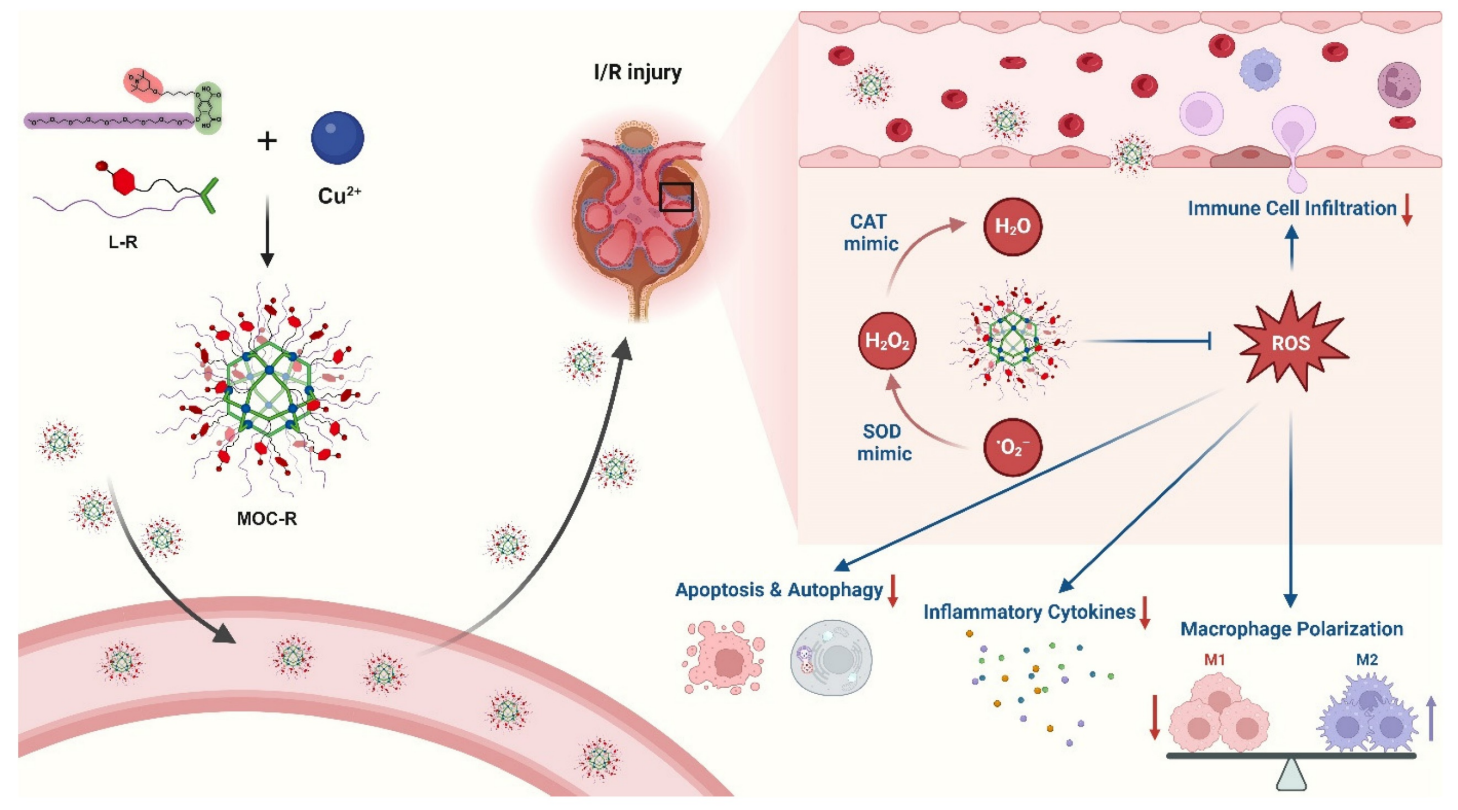
Schematic illustration of preparation of precise and ultrasmall MOC-R cascade nanozyme and its application in treating renal I/R injury. Created with BioRender.com.

In this study, we synthesized a novel precise and ultrasmall radical MOC nanozyme with cuboctahedral topology and good water solubility, MOC-R, through robust coordination between copper ions and functional isophthalic acid, incorporating oligoethylene glycol and 2,2,6,6-tetramethylpiperidin-1-oxyl (TEMPO) 38,39. The mechanistic investigations demonstrated that TEMPO and the copper component drive the mimicry of superoxide dismutase (SOD) 40,41 and catalase (CAT) 42,43 activities, respectively, resulting in exceptional antioxidant activity due to their synergistic effects. *In vitro* and *in vivo* experiments revealed that this synergy provides significant radical scavenging and anti-inflammatory benefits by reducing immune cell infiltration, promoting macrophage polarization to the M2-like phenotype, limiting inflammatory cytokine release, and inhibiting excessive autophagy and apoptosis (Scheme 1). In summary, this atomically precise and ultrasmall cascade antioxidant nanozyme demonstrates exceptional stability, biocompatibility, ROS scavenging capacity, and anti-inflammatory performance. These properties are expected to significantly advance structure-activity studies and facilitate clinical translation.

## Results and Discussion

### Preparation and characterization of MOC-R nanozyme

**Figure 1:**
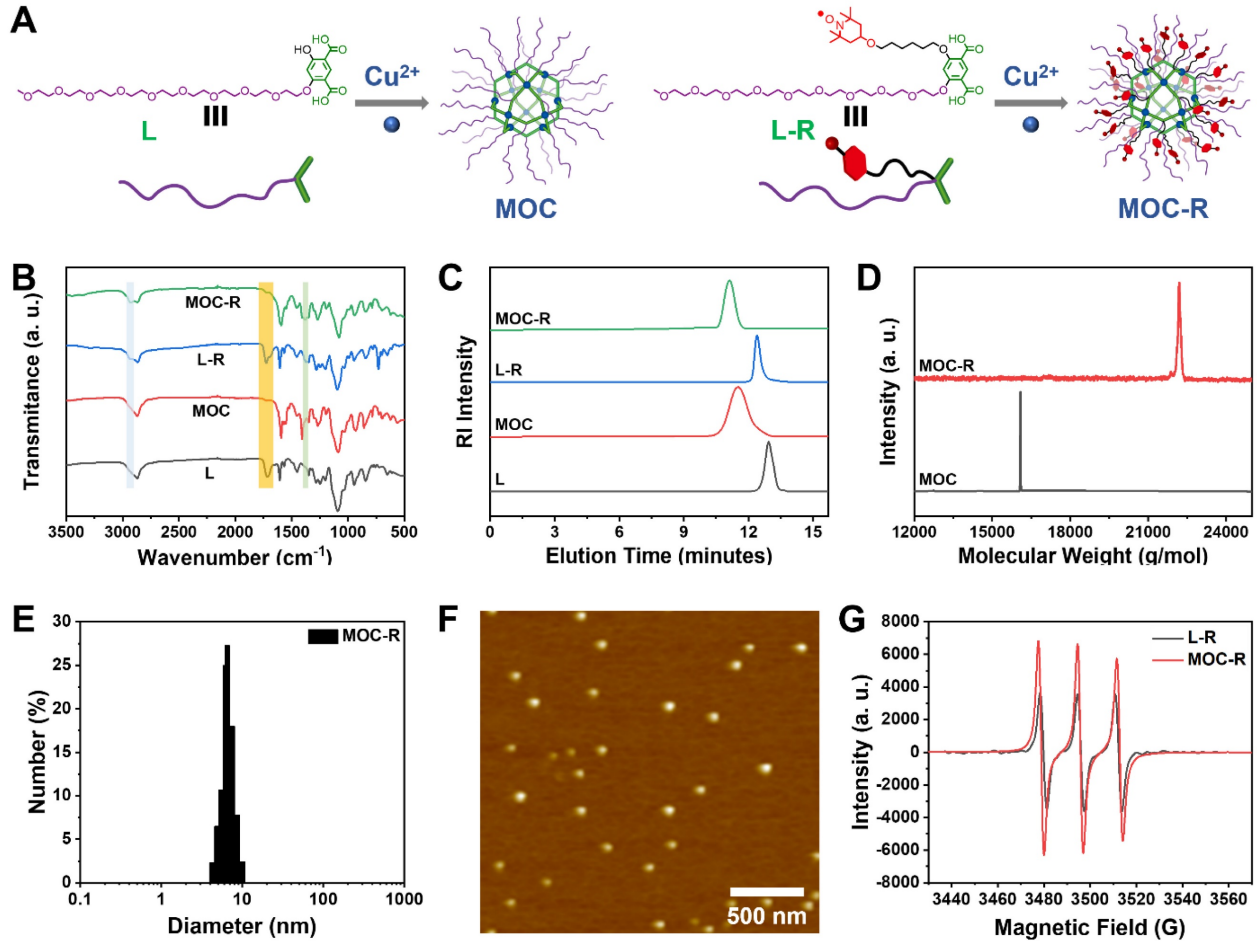
** Preparation and Characterization of MOC-R cascade nanozyme. (A)** The structures of ligands, MOC, and MOC-R. **(B)** The FT-IR spectra of ligands, MOC, and MOC-R. **(C)** The SEC profiles of the ligands, MOC, and MOC-R in THF. **(D)** The MALDI-TOF mass spectra of MOC and MOC-R.** (E)** The DLS plot of MOC-R in PBS. **(F)** The AFM image of MOC-R. Scale bars represent 500 nm. **(G)** The EPR curves of L-R and MOC-R.

The detailed synthetic procedures and structural characterizations are provided in the Figure 1A and supporting information (Scheme S1, and Figures S1-S12). To investigate the source of enzyme-like activity, water-soluble MOC lacking radical moieties is synthesized from ligand L. Analysis of the ^1^H-NMR spectra for L-R and MOC-R (Figure S13) revealed that all peaks for MOC-R were broadened and shifted to lower fields compared to those of L-R, which indicate the coordination between L-R and Cu^2+^, similar to previously reported results 24. Specifically, two characteristic peaks at 8.41 and 6.37 ppm, attributed to the benzene ring in L-R, shifted to 8.65 and 6.73 ppm in MOC-R. Fourier transform infrared (FT-IR) spectra showed that both ligand L and L-R exhibited a carboxylic acid signal at 1700 cm^-1^. This signal disappeared in MOC and MOC-R due to coordination effects (Figure 1B). Additionally, characteristic signals for TEMPO at 2940 cm^-1^ and 1387 cm^-1^ appeared in L-R and MOC-R but were absent in L and MOC. These NMR and FT-IR results confirm the successful coordination between the ligands and Cu^2+^ to form MOC and MOC-R.

Typically, coordination between isophthalic acid and copper ions results in the formation of a cuboctahedral MOC with a stoichiometric Cu_24_L_24_ composition. Due to their large molecular weights, size exclusion chromatography (SEC) was employed to analyze the molecular weight, stability, and polydispersity of the ligands and MOCs. SEC plots revealed single sharp peaks at approximately 12.90 min for L and 12.42 min for L-R, and peaks at 11.62 min for MOC and 11.15 min for MOC-R (Figure 1C). The shorter elution times for MOC and MOC-R likely reflect their larger hydrodynamic volumes compared to the ligands. The molecular weights of MOC and MOC-R, as determined from the SEC plots, were 15763 g/mol and 20867 g/mol, respectively, closely matching the theoretical values of 16084 g/mol and 22189 g/mol. Furthermore, MOC and MOC-R exhibited narrow polydispersity indices of 1.13 and 1.06, respectively, indicating high uniformity, homogeneity, and stability. These molecular weights were also confirmed by matrix-assisted laser desorption ionization time-of-flight (MALDI-TOF) mass spectrometry (Figure 1D), where peaks at 16084 and 22189 g/mol were observed, consistent with the theoretical values, confirming the formation of cuboctahedral MOC and MOC-R.

Benefiting from the surface OEG moieties, the water solubility of MOC-R is about 2.5 mg/mL. The size and surface charge of MOC-R were determined using dynamic light scattering (DLS) and zeta potential measurements for subsequent biological applications. MOC-R exhibited a hydrated particle size of approximately 7.0 nm with a narrow polydispersity of 0.18 (Figure 1E) and a slightly negative charge of -3.5 mV, suggesting good biosafety. The DLS results of MOC-R after being dissolved in PBS or fetal bovine serum (FBS) for one day are consist with above value, indicating its structural stability (Figure S14). Atomic force microscopy (AFM) was used to visualize the morphology and size of MOC-R (Figure 1F). The height of MOC-R was approximately 4.5 nm, showing high uniformity, which aligns with the theoretical size of the cuboctahedral MOC and confirms its structural stability, uniformity, and ultrasmall size. Electron paramagnetic resonance (EPR) spectroscopy was employed to examine the radical characteristics of L-R and MOC-R (Figure 1G). The EPR spectrum of L-R, showing three consistent peaks, was identical to that of unmodified TEMPO, indicating that the radical properties were retained. MOC-R also displayed intense radical signals, confirming the presence of radical characteristics after the formation of MOC-R. The UV-vis spectra of MOC and MOC-R in PBS (0.2 mg/mL) show negligible absorption in 500~650 nm, suitable for the further enzyme activity evaluation (Figure S15).

### SOD-like and CAT-like activities of MOC-R nanozyme

**Figure 2:**
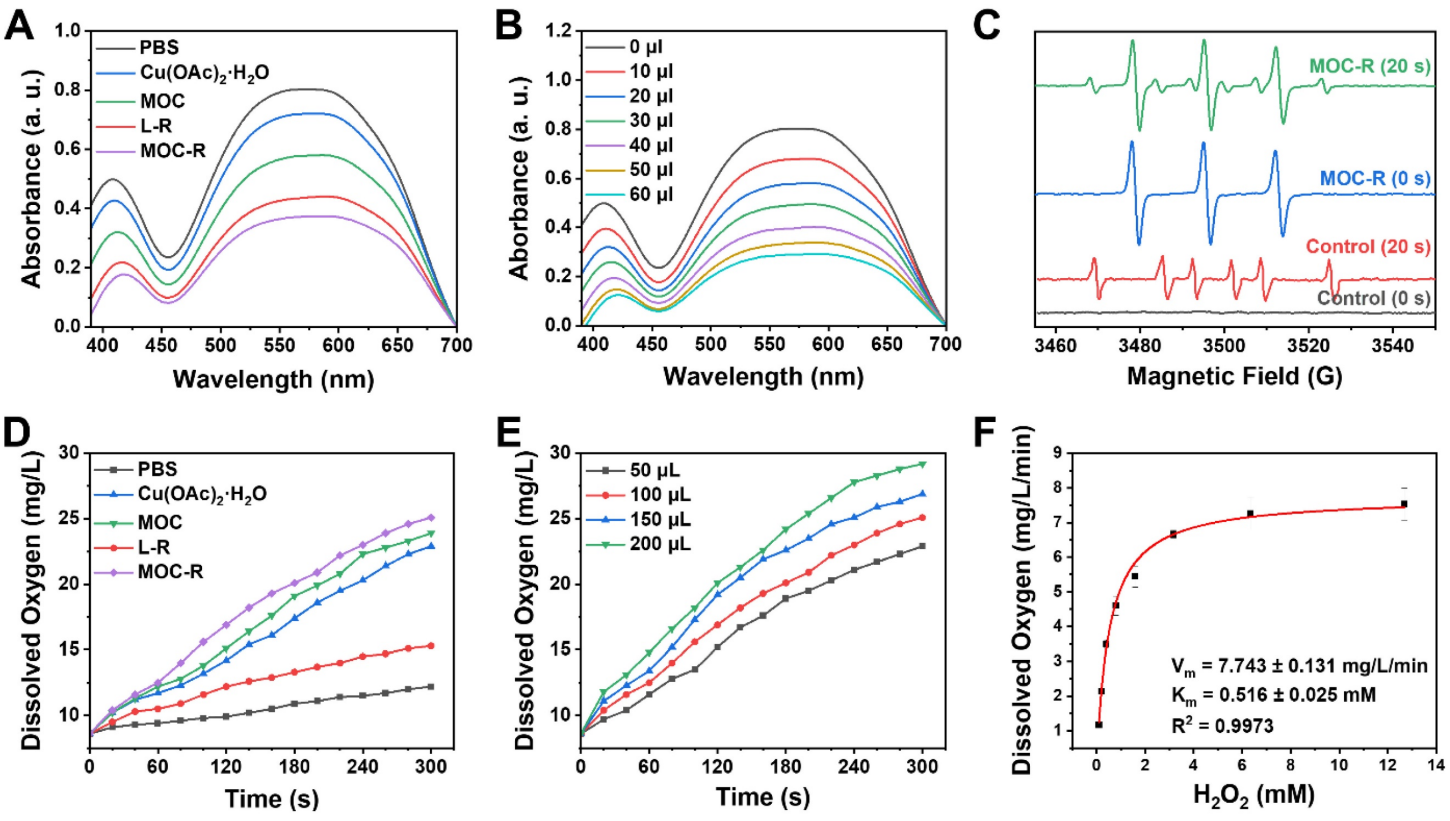
** The antioxidant enzyme-like activities of MOC-R nanozyme. (A)** The SOD-like activities of different groups. **(B)** Correlation between the amount of MOC-R and its SOD-like activity. **(C)** EPR detection of ·O_2_^-^ signals under varying conditions. **(D)** Kinetics of O_2_ generation for CAT-like activity of MOC-R. **(E)** Impact of MOC-R amount on its CAT-like activity. **(F)** Kinetic analysis and Michaelis-Menten fitting of the CAT-like activity of MOC-R.

After fully characterizing the MOC-R nanozyme, its atomically precise and ultrasmall structure and radical features prompted us to explore its enzyme-like activities. Given that scavenging superoxide anions (·O_2_^-^) is crucial in anti-ROS cascade reactions and considering the established activity of the TEMPO, we initially investigated its SOD-like activity by monitoring ·O_2_^-^ elimination. Results from nitrotetrazolium blue chloride (NBT) 44, a redox indicator for detecting ·O_2_^-^, demonstrated that MOC-R and its components exhibited notable SOD-like activity compared to the PBS control group (Figure 2A). Specifically, Cu(OAc)_2_·H_2_O, L-R, and MOC all displayed evident SOD-like activity, with the radical L-R showing superior performance. Interestingly, the SOD-like activity of the assembled MOC was significantly higher than that of Cu(OAc)_2_·H_2_O, likely due to the active species in MOC being the Cu_2_(COO^-^)_4_ paddlewheel cluster rather than free copper ions 45,46. Notably, the integrated MOC-R exhibited the most pronounced SOD-like characteristics, indicating that the formation of MOC-R enhanced its SOD-like activity, primarily due to the TEMPO moieties and assisted by the internal copper clusters. Further investigation revealed that the SOD-like performance of MOC-R was concentration-dependent, with increased activity observed as the concentration of MOC-R increased (Figure 2B). Additionally, EPR spectrometry confirmed the ·O_2_^-^ scavenging capability of MOC-R nanozymes, as evidenced by a significant reduction in EPR signals upon the addition of MOC-R despite the signal overlap of TEMPO within MOC-R and ·O_2_^-^ (Figure 2C). The SOD-like specific activity of MOC-R is calculated to be 3.16 U/mg (Figure S16) and EC_50_ is about 108 μg/mL.

Following the disproportionation of ·O_2_^-^, hydrogen peroxide (H_2_O_2_) was further catalyzed by catalase (CAT) into water and oxygen. We assessed H_2_O_2_ consumption and oxygen generation using a dissolved oxygen detector to validate the CAT-mimicking activities of MOC-R nanozyme (Figure 2D) 47. Cu(OAc)_2_·H_2_O, MOC, and MOC-R all exhibited significant CAT-like activities, with MOC showing better activity and MOC-R demonstrating the highest. The superior CAT-like activity of the assembled MOC compared to dissolved Cu(OAc)_2_·H_2_O may also stem from differences in active species. The MOC-R, integrating both active radicals and copper clusters 48, enhanced CAT-like activity, primarily attributed to the active copper clusters and assisted by the external TEMPO moieties. The elimination rates for MOC-R were concentration-dependent, with higher quantities of MOC-R accelerating H_2_O_2_ catalysis (Figure 2E). Moreover, CAT-like kinetics, determined by oxygen generation, confirmed that MOC-R nanozyme exhibited excellent CAT-like activity in converting H_2_O_2_ into water and oxygen in a concentration-dependent manner (Figure S17). To further determine the CAT-like activity of MOC-R, a kinetic analysis was carried out. The Michaelis-Menten constant (Km) value of MOC-R with O_2_ as substrate was calculated by the Michaelis-Menten equation to be 0.516 mM, and the maximum velocity (Vm) value was 7.743 mg/L/min (Figure 2F). Collectively, MOC-R demonstrated excellent SOD- and CAT-like activities *in vitro* benefiting from the synergism between external TEMPO moieties and internal copper clusters. Further, we also estimated the radical scavenging ability of MOC-R (Figure S18-19). The inhibition of hydroxyl radicals and ABTS radicals of MOC-R were larger than 80% at concentrations of 0.40 and 0.66 mg/mL, respectively.

### The *in vitro* cellular protective and ROS-scavenging efficacy of MOC-R nanozyme

**Figure 3:**
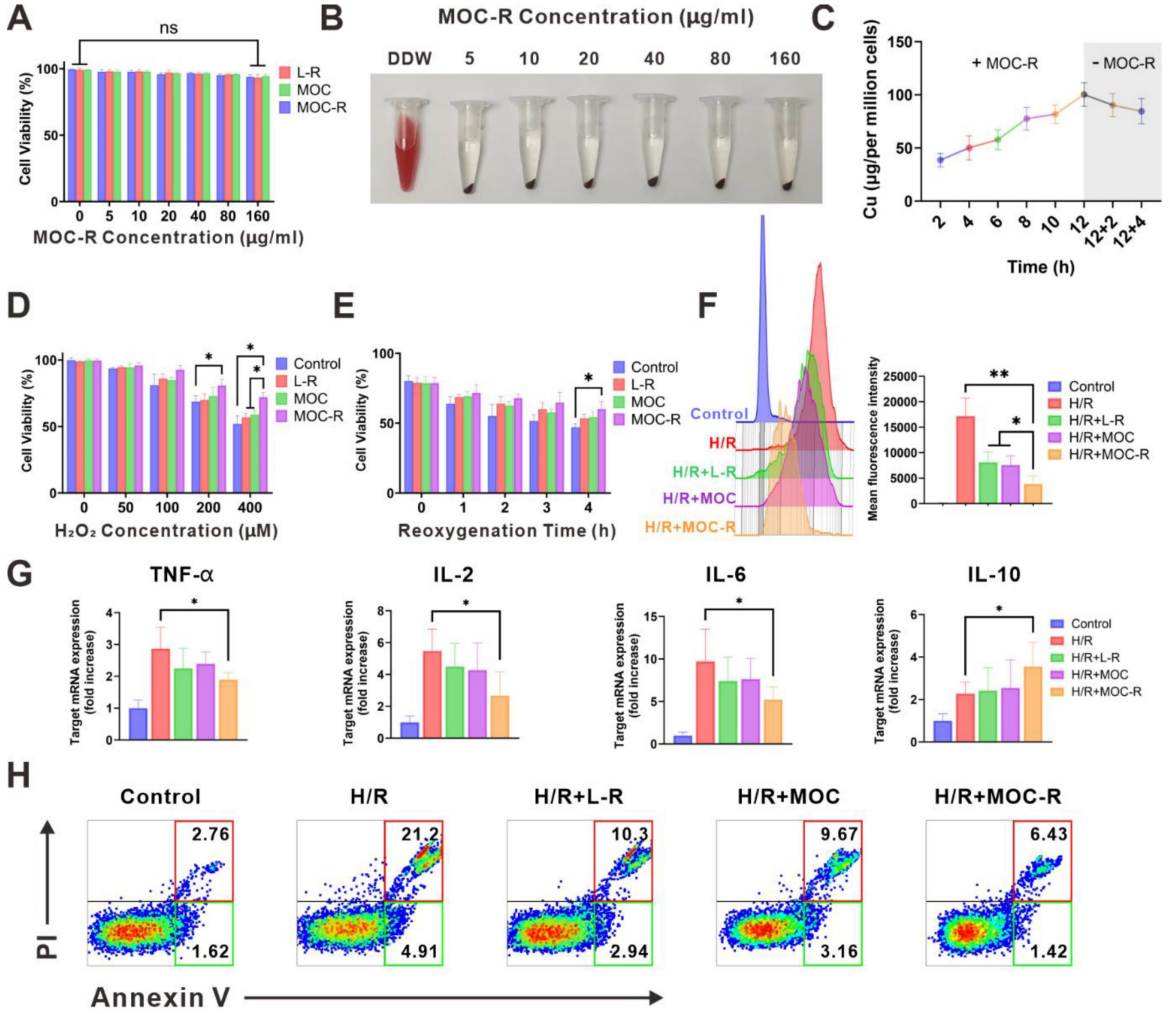
** The *in vitro* cellular protective and ROS-scavenging efficacy of MOC-R nanozyme. (A)** The impact of MOC-R on HK-2 cell viability assessed using the MTT assay. **(B)** Assessment of hemolytic activity of double-distilled water (DDW) or MOC-R on HK-2 cells. **(C)** Cellular uptake of MOC-R in HK-2 cells. **(D)** MTT assay of MOC-R on H_2_O_2_ treated HK-2 cells. **(E)** MTT assay of MOC-R on H/R assaulted HK-2 cells. **(F)** Flow cytometry and fluorescence intensity analysis to evaluate the influence of various treatments on HK-2 cells post H/R. **(G)** The mRNA levels of TNF-α, IL-2, IL-6, and IL-10 in HK-2 cells following H/R. **(H)** Flow cytometry analysis using Annexin V/PI staining to determine apoptosis of HK-2 cells after H/R. Data are presented as mean ± s.d. ns *p >* 0.05, * *p <* 0.05, ** *p <* 0.01.

To explore the antioxidant potential of MOC-R in biomedical applications, we focused on its biocompatibility firstly. Initial assessments included methyl thiazolyl tetrazolium (MTT) 49 assays and hemolysis tests at the cellular level. Human kidney-2 (HK-2) cells demonstrated a survival rate exceeding 90% across various MOC-R concentrations (0-160 µg/mL), indicating excellent biocompatibility (Figure 3A). Hemolysis rates remained exceptionally low, even at high MOC-R concentrations (160 µg/mL), further confirming its minimal toxicity to erythrocytes (Figure 3B). Cellular uptake of MOC-R was assessed using inductively coupled plasma mass spectrometry (ICP-MS), revealing increased MOC-R content in HK-2 cells with prolonged incubation (Figure 3C). The ultrasmall size of MOC-R and its non-robust Cu_2_(COO^-^)_4_ cluster facilitated gradual release from the cells in either integrated or decomposed forms.

Subsequently, we evaluated the cellular protective capacity of MOC-R nanozyme against stressors induced by H_2_O_2_ or hypoxia/reoxygenation (H/R) exposure (Figure 3D-E). MTT assay results showed a significant reduction in HK-2 cell viability with H_2_O_2_ treatment or H/R exposure compared to controls. MOC-R treatment, however, notably mitigated these adverse effects and preserved cell viability, highlighting its potential as a protective agent against oxidative stress. To elucidate MOC-R's mechanism, we assessed its *in vitro* antioxidant efficacy using the DCFH-DA 50 probe through flow cytometry (Figure 3F). H/R treatment elevated ROS levels in HK-2 cells, with L-R, MOC, and MOC-R all demonstrating ROS scavenging abilities. MOC-R proved significantly more effective than the other groups, underscoring its potent antioxidant performance. We subsequently quantified the mRNA expression levels of inflammation-related cytokines in HK-2 cells following H/R injury. Our results demonstrated that the mRNA expression of pro-inflammatory cytokines (TNF-α, IL-2, and IL-6) significantly increased after H/R injury, while the mRNA expression of the anti-inflammatory cytokine IL-10 significantly decreased. As expected, treatment with the MOC-R nanozyme markedly reversed these changes (Figure 3G). Moreover, Annexin V/PI staining revealed that MOC-R significantly reduced H/R-induced apoptosis in HK-2 cells, indicating that MOC-R not only attenuates inflammation but also enhances cell survival under conditions of oxidative stress (Figure 3H).

### MOC-R nanozyme protected against renal I/R injury

**Figure 4:**
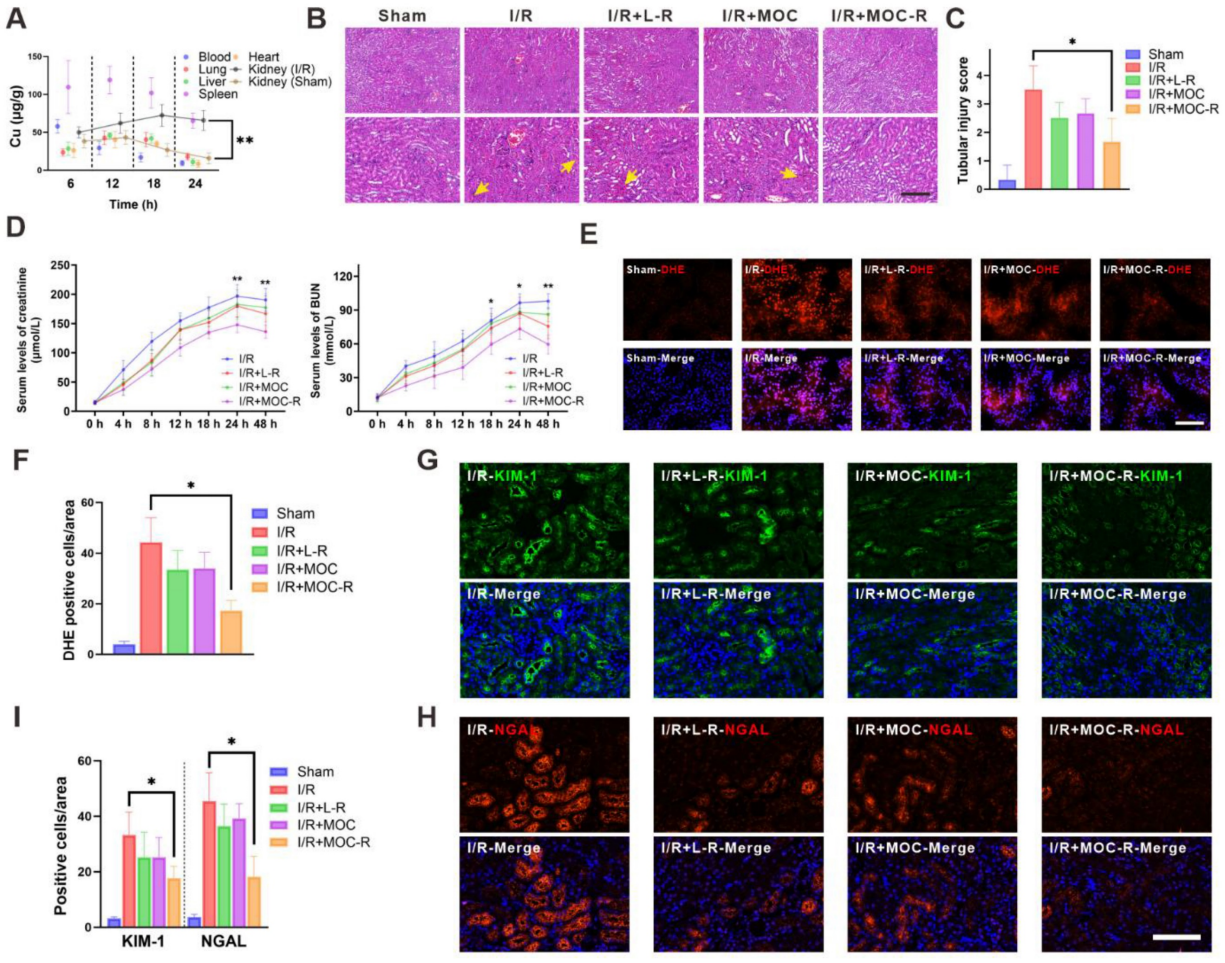
** The protective effect of MOC-R nanozyme against I/R injury. (A)** The biodistribution of MOC-R in diverse tissues as indicated by the copper concentration. **(B)** Representative Hematoxylin and eosin staining of the kidney at 24 h post reperfusion (10× and 20×). Vascular congestions are indicated by arrows. Scale bars represent 100 μm. **(C)** Assessment of kidney injury based on tubular injury scores. **(D)** Serum creatinine and BUN levels in renal I/R injury. **(E)** Representative immunofluorescence images of DHE expression at 24 h post reperfusion. Scale bars represent 100 μm. **(F)** The count of the DHE positive cells. **(G)** Representative immunofluorescence images showing KIM-1 expression at 24 h post reperfusion. **(H)** Representative immunofluorescence images showing NGAL expression at 24 h post reperfusion. Scale bars represent 100 μm. **(I)** The count of the KIM-1 and NGAL positive cells. Data are presented as mean ± s.d. * *p <* 0.05, ** *p <* 0.01.

Firstly, the *in vivo* biocompatibility of MOC-R nanozyme was evaluated by intravenous injection of twice the usual dose. Two days later, the major organs were harvested and underwent histological analysis via H&E staining (Figure S20) 51. The outcomes demonstrated that the histological injury of all groups of mice were difficult to detect, thereby indicating that MOC-R nanozyme did not inflict any conspicuous damage on the mice, ascertaining the favorable *in vivo* biocompatibility of MOC-R. Next, ICP-MS was employed to analyze the biodistribution at diverse time points subsequent to the injection of MOC-R solution, serving as an approach to explore the protective mechanism of MOC-R nanozyme in renal I/R injury (Figure 4A). Evidently, the concentration of MOC-R nanozyme in the bloodstream decreased over time, and the spleen and I/R kidney displayed the highest levels of MOC-R accumulation among the organs analyzed. The accumulation of MOC-R in the I/R-injured kidney can be attributed to the increased vascular permeability resulting from I/R injury, which disrupts endothelial cell junctions and triggers the release of pro-inflammatory mediators, thereby compromising the integrity of the vascular barrier. Moreover, owing to the ultrasmall particle size of MOC-R, a considerable portion had been metabolized within 24 h across all the examined tissues.

Oxidative stress arises from an imbalance between the production of ROS and the body's ability to detoxify these reactive intermediates or repair the resulting damage. This imbalance can lead to cellular dysfunction, tissue damage, and ultimately, organ failure. We therefore evaluated the protective role of MOC-R nanozyme in renal I/R injury. All mice in the study were subjected to 30 min of ischemia. Renal tissues of mice in the I/R group all manifested substantial tubular necrosis and impairment after I/R injury. In contrast, mice treated with the L-R and MOC groups exhibited lessened tubular necrosis and damage after I/R injury; however, the effect was rather low. This was likely because molecular L-R was so prone to be cleared out and difficult to exert its effect. Nevertheless, mice treated with MOC-R exhibited significant alleviation of tubular and renal damage following I/R injury, as evidenced by minimal loss of brush border, limited intratubular debris, and reduced glomerular congestion. In contrast, the other groups displayed more pronounced tubular necrosis (Figure 4B-C). The renal protective effects of MOC-R were further examined by assessing creatinine and blood urea nitrogen (BUN) levels following I/R injury (Figure 4D). The outcomes indicated that both creatinine and BUN levels progressively increased and peaked at 24 h after I/R injury, then began to decline after 24 h. In contrast to the I/R group, L-R or MOC treatments slightly reduced the levels of creatinine and BUN, while the levels dropped significantly after MOC-R treatment. Given that MOC-R nanozyme exhibited its anti-oxidative capability *in vitro*, we next verified whether the amelioration of oxidative stress constitutes the underlying mechanism through which MOC-R nanozyme confers renal protection against I/R injury. By utilizing dihydroethidium (DHE), an intracellular superoxide indicator capable of being oxidized by superoxide to yield a red fluorescent product, we discovered that the fluorescence intensity of DHE-stained kidney tissue was augmented subsequent to I/R insult, indicating a high degree of ROS generation (Figure 4E-F). This increase was mitigated by L-R or MOC treatment, and was further diminished by MOC-R nanozyme treatment, demonstrating that MOC-R nanozyme inhibited I/R-induced oxidative stress in the kidneys 52,53.

Kidney injury molecule-1 (KIM-1), a biomarker of kidney injury, is expressed at an extremely low level in healthy kidneys and promptly upregulated following damaging events 54. Neutrophil gelatinase-associated lipocalin (NGAL), a small protein belonging to the lipocalin family, is physiologically produced at a minimal level but rises under harmful circumstances. We then tested KIM-1 and NGAL using immunofluorescence to evaluate the biomarkers expression in injured kidneys. Subsequently, we employed immunofluorescence to visualize KIM-1 and NGAL for evaluating the molecular changes occurring in injured kidneys (Figure 4G-I). The results revealed that the expression of NGAL and KIM-1 was significantly up-regulated in I/R mice. However, no significant effect was observed after treatment with L-R and MOC, and a marked decrease in the expression of NGAL and KIM-1 was detected after treatment with MOC-R, indicating that MOC-R significantly changed the expression patterns of I/R induced renal damage.

### MOC-R nanozyme restricted I/R induced inflammatory milieu and programmed cell death

**Figure 5:**
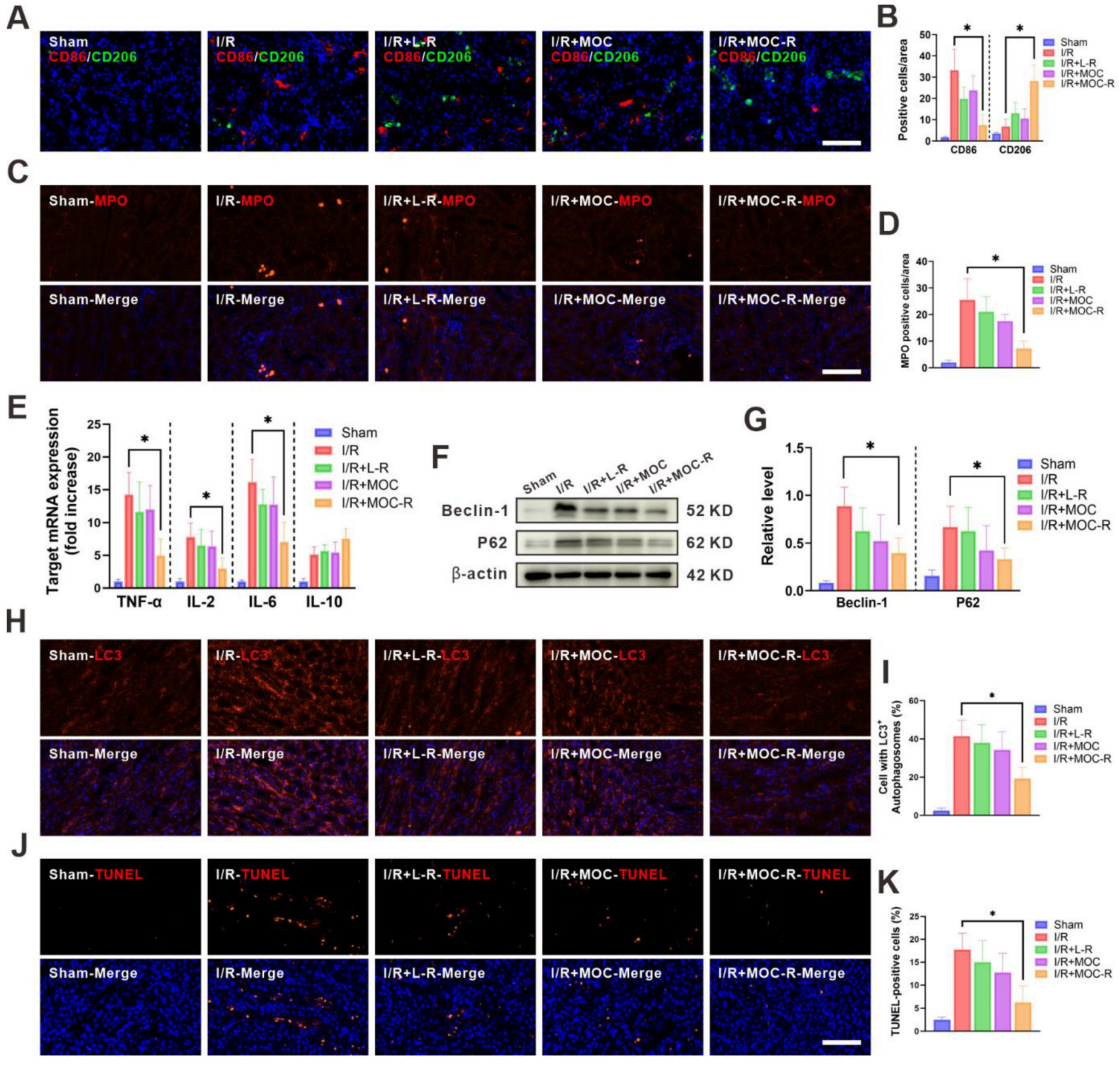
** MOC-R nanozyme restricted I/R induced inflammatory milieu and programmed cell death. (A)** Representative immunofluorescence images showing CD86 and CD206 expression at 24 h post reperfusion. Scale bars represent 100 μm. **(B)** The count of the CD86 and CD206 positive cells. **(C)** Representative immunofluorescence images showing MPO expression at 24 h post reperfusion. Scale bars represent 100 μm. **(D)** The count of the MPO positive cells. **(E)** The mRNA levels of TNF-α, IL-2, IL-6, and IL-10 in renal tissue. **(F)** Expression of Beclin-1 and P62 detected by western blotting assay. **(G)** The relative densities of the bands in each lane were analyzed and normalized to β-actin. **(H)** Representative immunofluorescence images showing LC3 expression at 24 h post reperfusion. **(I)** The count of the cells with LC3 positive autophagosomes. **(J)** Representative immunofluorescence images showing TUNEL expression at 24 h post reperfusion. Scale bars represent 100 μm. **(K)** The count of the TUNEL positive cells. Data are presented as mean ± s.d. * *p <* 0.05.

ROS are essential for the induction and maintenance of M1 macrophage polarization. Numerous studies have highlighted the role of ROS in activating both the NF-κB and p38/MAPK signaling pathways, which in turn promote pro-inflammatory gene expression in macrophages 55,56. Immunofluorescence assays demonstrated that I/R insult augmented the expression of the M1 macrophage phenotype (CD86), yet L-R or MOC treatment marginally reversed the M1 phenotype to the M2 macrophage marker (CD206) 57. Intriguingly, the expression of CD86 substantially declined concomitant with a notable increase in the expression of CD206 following MOC-R nanozyme treatment, which was precisely the converse of that in I/R kidneys (Figure 5A-B). Given the significant role of M2 macrophages in kidney fibrosis, we utilized Masson's trichrome staining to assess collagen deposition in renal tissues and evaluate the long-term effects of MOC-R on renal fibrosis (Figure S21). Our results indicated that MOC-R did not increase collagen deposition, suggesting it does not promote fibrotic changes. Additionally, neutrophil infiltration after reperfusion was quantified by immuno-fluorescence assays (with positive cells indicated by red fluorescence normalized to DAPI-stained nuclei). A considerable increase in neutrophils was noted in I/R-injured kidney compared to the sham group. Neither L-R nor MOC treatment proved effective in lowering neutrophil levels, a propensity that was effectively alleviated by treatment with MOC-R nanozyme, which significantly decreased neutrophil levels (Figure 5C-D). We then measured the mRNA expression of inflammatory-related cytokines in kidney tissues following I/R injury (Figure 5E), and discovered that the mRNA expression of proinflammatory cytokines (TNF-α, IL-2, and IL-6) significantly increased after I/R injury, while the MOC-R nanozyme markedly decreased them. In contrast, upon I/R injury, the mRNA expression of the anti-inflammatory cytokine (IL-10) declined, whereas the MOC-R nanozyme elevated it to a considerable extent, albert missing the significance. These findings demonstrated that the MOC-R nanozyme can not only inhibit the polarization of macrophages to the M1 phenotype and facilitate its polarization to the M2 phenotype but also enhance the suppression of the I/R-induced inflammatory response.

Autophagy is a regulated cellular process of self-degradation and is typically regarded as an inducible adaptive response to cellular stress. However, dysregulation or excessive activation of autophagy, such as that occurring during I/R injury, triggers autophagic cell death without caspase involvement. Herein, western blotting was performed to detect the expression levels of autophagy-related proteins (Beclin-1 and P62) in order to evaluate autophagic flux in kidneys following I/R injury (Figure 5F). As expected, MOC-R treatment significantly reduced the expression levels of Beclin-1 and P62 (Figure 5G). In line with the western blotting data, immunostaining targeting LC3 manifested a pronounced increase in the number of autophagosomes within the kidney tissue after I/R and a decrease subsequent to MOC-R nanozyme treatment. However, L-R or MOC treatment had no effect on the autophagy level in I/R kidneys (Figure 5H-I). Apoptosis is a tightly-regulated ATP-dependent process of programmed cell death that is activated by hypoxia due to ischemia and during ROS generation in reperfusion. The level of cellular apoptosis in I/R kidney was assessed using the TUNEL assay in this study (Figure 5J-K). Our findings indicated that the proportions of TUNEL-positive cells were significantly lower in mice treated with MOC-R than in the control group. These results indicated that the MOC-R nanozyme modulated the anti-inflammatory response by alleviating oxidative stress, thereby reducing programmed cell death in kidneys subjected to I/R injury.

## Conclusion

In this study, an atomically precise, ultrasmall, and water-soluble cascade antioxidant nanozyme was constructed based on a metal organic cage integrating active organic radicals and metal clusters. Through the assembly of ligands containing radicals and copper ions, the water-soluble MOC-R could be obtained in a high yield. The particle size of MOC-R is approximately 7.0 nm, and it is negatively charged; these properties of MOC-R are beneficial for the clinical application of antioxidants *in vivo*. The MOC-R compound not only demonstrates SOD-like activity and CAT-like activity, but also showcases remarkable potential in the field of antioxidant therapy. Its SOD-like activity is primarily attributed to the presence of TEMPO, a key component that contributes to its free radical scavenging abilities. Meanwhile, the CAT-like activity of MOC-R arises from the unique properties of its copper clusters, which enable it to efficiently neutralize ROS. As a result, this cascading nanozyme is capable of efficiently scavenging harmful ROS, particularly •O_2_^-^ and H_2_O_2_, under stressful conditions. MOC-R also displays excellent biocompatibility and significant ROS-scavenging ability at both *in vitro* and *in vivo* levels, exerting exceptional renoprotective potency by influencing the anti-inflammatory status of the immune response and regulating programmed cell death. In conclusion, the multifaceted capabilities of MOC-R make it a compelling candidate for further exploration in biomedical research aimed at combating oxidative stress-related disorders.

## Materials and Methods

### SOD-like activity

SOD-like activity was determined using the nitrogen blue tetrazolium (NBT) method. Under UV irradiation, riboflavin and methionine can react to produce ·O_2_^-^, which reduces NBT to generate blue methylhydrazone with a maximum absorption wavelength of 560 nm. However, SOD can scavenge ·O_2_^-^ and inhibit the generation of methylhydrazone. Therefore, the higher the SOD-like activity, the lower the amount of the reduction product (methylhydrazone), and the lower the absorbance at 560 nm. Thus, the decrease in absorbance at 560 nm confirms the role of SOD-like activity. The PBS solution of samples (2 mg/mL, 90 μL) were added to the mixture of NBT (1 mg/mL, 0.5 mL), Met (30 mg/mL, 0.5 mL), EDTA-Na_2_ (0.4 mg/mL, 0.3 mL), riboflavin (1 mg/mL, 0.5 mL) and PBS solution (pH = 7.4, 2 mL) and incubated together under constant UV irradiation for 60 s to test the SOD-like activity. The experiments were divided into five groups due to the different samples: (1) PBS; (2) Cu(OAc)_2_∙H_2_O; (3) L-R; (4) MOC; (5) MOC-R. The concentration-dependent SOD-like activities were also determined by varying the concentrations of MOC-R under the same conditions and incubated under UV irradiation for 60 s.

To further investigate the SOD-like activity of MOC-R, we employed EPR spectroscopy for a more intuitive assessment. 5,5-Dimethyl-1-pyrroline N-oxide (DMPO) was used as a potent free radical trapping agent, capable of forming spin adducts upon interaction with the ·O_2_^-^ radical. The spin adducts, which can be detected by EPR, provide a direct measurement of the ·O_2_^-^ radical concentration in the reaction system. The reaction mixture consisted of the following components: Met (130 mM, 0.25 mL), EDTA-Na_2_ (0.1 mM, 0.25 mL), H_2_O (0.20 mL), MOC-R (2 mg/mL, 0.05 mL), riboflavin (0.02 mM, 0.25 mL), DMPO (100 mM, 70 μL), and phosphate buffer (pH 7.4, 50 mM, 1.68 mL). The mixture was thoroughly mixed and then placed into a nuclear magnetic resonance tube for subsequent UV irradiation at two time points: 0 s and 20 s, followed by EPR spectrum analysis.

### •O_2_^-^ inhibition rate

Mixture of NBT (1 mg/mL, 0.4 mL), Met (30 mg/mL, 0.4 mL), EDTA-Na_2_ (0.4 mg/mL, 0.25 mL), riboflavin (1 mg/mL, 0.4 mL) and PBS solution (pH = 7.4, 1.85 mL) to obtain the detection working solution. Subsequently, MOC-R solutions (0.35-2.45 mg/mL, 200 μL) or PBS (200 μL) was added to above detection working solution and immediately irradiated it with ultraviolet light. Record the changes in absorbance between the sample and blank, calculate the inhibition rate, and obtain the enzyme activity unit.

Inhibition rate (%) = (A_b_ - A_s_)/A_b_ × 100

where A_b_ is the absorbance of the blank and A_s_ is the absorbance of the sample.

### CAT-like activity

CAT-like activity was determined using a dissolved oxygen meter. Oxygen produced was measured at room temperature by using a specific oxygen electrode on a split dissolved oxygen meter (SW9403). Sample (2 mg/mL, 100 μL) and H_2_O_2_ solution (30%, 200 μL) was added to water (14.94 mL), and the concentration (in mg/L) of produced oxygen was recorded from 0 to 5 min. To assess the CAT-like activity, five samples were set up: (1) PBS; (2) Cu(OAc)_2_∙H_2_O; (3) L-R; (4) MOC; and (5) MOC-R. In addition, different amounts (50, 100, 150, and 200 μL) of MOC-R solution (2 mg/mL) were added to the final volume of the buffer solution (15 mL), and their corresponding CAT-like activity was determined.

### CAT-like kinetics

A series of solutions of hydrogen peroxide (0.10-12.68 mM in PBS, 25 mL) was prepared, then MCO-R solution (1 mg/mL in PBS, 0.10 mL) was added. The dissolved oxygen in the above mixture was immediately monitored for 5 min using a dissolved oxygen analyzer. The corresponding reaction rate and hydrogen peroxide concentration were then fitted to the Michaelis-Menten equation to obtain the relevant reaction kinetics parameters.

### Hydroxyl radical scavenging

The reaction mixture contains FeSO_4_ (6 mM, 0.1 mL), hydrogen peroxide (6 mM, 0.1 mL), and sample (0.15-2.4 mg/mL in PBS, 0.1 mL). The mixture was incubated at 37 ºC for 10 min, covered with salicylic acid ethanol solution (6 mM, 0.1 mL), and then further incubated at 37 ºC for 30 min. Measure the mixture at 510 nm using an enzyme-linked immunosorbent assay reader. Ultrapure water was used as a blank instead of the sample.

Hydroxyl radical scavenging activity (%) = (A_b_ - A_s_)/A_b_ × 100

where A_b_ is the absorbance of the blank and A_s_ is the absorbance of the sample.

### ABTS radical scavenging activity

The solutions of ABTS (7.0 mM in DDW), potassium persulfate (9.4 mM in DDW), and MOC-R (0.25-2.0 mg/mL in PBS) were prepared firstly. Mixing the solutions of ABTS and potassium persulfate in equal volume at room temperature in the dark for 12 h and then diluted for 20 times with PBS to obtain the ABTS working solution. Add ABTS working solution (0.2 mL) and samples (0.15-2.4 mg/mL in PBS, 0.1 mL) to each well on a 96 well plate, incubate for 30 min, and measure the absorbance at 734 nm using an enzyme-linked immunosorbent assay reader.

ABTS radical scavenging activity (%) = (A_b_ - A_s_)/A_b_ × 100

where A_b_ is the absorbance of the blank and A_s_ is the absorbance of the sample.

### Cytotoxicity assay

To assess the cytotoxic effect of MOC-R, HK-2 (7000 cells/well) cells were added into a 96 well plate, and cultured at 37 °C for 12 h. Different concentrations (0, 5, 10, 20, 40, 80, 160 μg/mL, 10 μL) of MOC-R solution in PBS were added in cells and cultured for another 24 h. Afterwards, the cells were gently washed by PBS (pH 7.4) for three times. The Thiazolyl Blue Tetrazolium Bromide (MTT) solution (Beyotime, Shanghai) was added to each well individually and incubated for 4 h in the dark. Subsequently, the cell supernatant was collected using a micropipettor, followed by addition of 150 μL of DMSO. After thorough agitation for 10 min to completely dissolve MTT crystals, cell viability was assessed using a microplate reader at absorbance wavelengths of 570 nm.

### Hemolysis assay

The whole blood of healthy mice was collected in a 2 mL EP tube, and the blood was centrifuged at 2000 rpm for 5 min to remove the upper serum and obtain erythrocytes. Then erythrocytes were washed three times with PBS, and 0.5 mL erythrocytes were diluted with 10 mL PBS. Then 0.5 mL of MOCs solution (5, 10, 20, 40, 80, 160 μg/mL), or DDW were added to 0.5 mL erythrocytes suspension, respectively. All sample tubes were kept at room temperature for 2 h and then centrifuged at 2000 rpm for 5 min. Finally, the experimental result was recorded by a camera.

### Cellular uptake

ICP-MS was used to accurately measure the uptake of MOC-R by HK-2 cells. In the *in vitro* cell uptake assay, HK-2 cells were inoculated into 10 mm tissue plates at a density of 10,000 cells/mL and cultured for 24 h. After 24 h, MOC-R (10 μg/mL, final concentration) was added to each tissue plate and cultured for 2, 4, 8, or 12 h.

In the *in vitro* cell retention assay, HK-2 cells were inoculated onto 10-mm tissue plates at a density of 10,000 cells/mL, and after 24 h of incubation, MOC-R (10 μg/mL, final concentration) was added to each tissue plate and incubated for another 12 h. After that, the medium was extracted and replaced with fresh medium (without MOC-R). Cells were subsequently collected at the “12 + 4” h and “12 + 8” h time points. After trypsinization and centrifugation steps, the cell pellet was washed three times with phosphate buffer solution. The samples were digested using a solution containing HNO_3_ (68%, 0.25 mL) and HCl (38%, 0.75 mL), and the digestion process lasted for 4 h at a temperature of 110 °C. After cooling, the sample was diluted with HCl (2%) to a volume of 10 mL. The copper concentration was then determined by ICP-MS.

### Intracellular ROS scavenging ability

Intracellular ROS levels were assessed using the fluorescent probe DCFH-DA. HK-2 cells (7000 cells per well) were cultured in 6-well microtiter plates at 37 °C for 12 h. To induce hypoxia, Na_2_S_2_O_4_ solution (2 mM) was added to the culture medium and the cells were placed in a CO_2_ incubator. The five experimental groups were designed as follows: (1) Control; (2) H/R; (3) H/R + L-R; (4) H/R + MOC; (5) H/R + MOC-R. The experimental groups (3-5) were added with samples (10 μg/mL, final concentration) and cultured at 37 °C for another 6 h. The results were analyzed using flow cytometry (FACSymphony, BD Biosciences, NJ).

### Real-time PCR analysis

The total cellular or renal RNA was extracted using TRIzol reagent (Invitrogen, Shanghai) according to the manufacturer's instructions. cDNA was transcribed using a Superscript III Reverse Transcriptase Kit (Invitrogen) and oligo d(T) (Applied Biosystems, Waltham, MA, USA). Quantitative RT-PCR analysis was performed with a SYBR RT-PCR kit (Takara, Tokyo) and the StepOne Real-Time PCR System (Applied Biosystems). All reactions were conducted in a 20 µL reaction volume in triplicate. The relative expression levels for a target gene were normalized against GAPDH. Primers used for RT-PCR analysis are as follows: TNF-α (5′-*AAG CCT GTA GCC CAC GTC GTA*-3′, 5′-*GGC ACC ACT AGT TGG TTG TCT TTG*-3′), IL-2 (5′-*CCA TGA TGC TCA CGT TTA AAT TTT*-3′, 5′-*CAT TTT CCA GGC ACT GGA GAT G*-3′), IL-6 (5′-*ACA ACC ACG GCCTTC CCT ACT T*-3′, 5′-*CAC GAT TTC CCA GAG AAC ATG TG*-3′), IL-10 (5′-*GCT TTA CTG ACT GGC ATG AG*-3′, 5′-*CGC AGC TCT AGG AGC ATG TG*-3′), and GAPDH (5′-*TGA CCA CAG TCC ATG CCA TC*-3′, 5′-*GAC GGA CAC ATT GGG GGT AG*-3′).

### Cellular apoptosis assay

An Annexin V-FITC/PI Apoptosis Detection Kit (Beyotime) was utilized to evaluate cell apoptosis. Cells were washed twice with cold PBS and subsequently harvested using trypsin without EDTA. The cells were resuspended in 195 μL of binding buffer. Next, 5 μL of Annexin V-FITC and 10 μL of propidium iodide staining solution were added, followed by incubation in an ice bath protected from light for 15 min. The percentage of apoptotic cells was quantified by flow cytometry (FACSymphony, BD Biosciences, NJ).

### Animal experimentation

Animal and Ethics Statement: C57BL/6 mice (female, 7-8 weeks old) were obtained from Shanghai Laboratory Animal Center, China. All animal experiments were performed in accordance with the guidelines of the National Institute of Health for the Care and Use of Laboratory Animals and approved by the Scientific Investigation Committee of Shanghai Changzheng Hospital (No. 202403013A).

### Pharmacokinetics analysis

The mice were sacrificed at the designated time points. Blood and all organs were collected, weighted and digested in a solution containing 0.25 mL HNO_3_ (68%) and 0.75 mL HCl (38%) for 12 h at 110 °C. After cooling, the samples were diluted with HCl (2%) to 10 mL. Cu contents were then detected by ICP-MS.

### Induced renal ischemia/reperfusion model

Mice were anesthetized with sodium pentobarbital (50 mg/kg, intraperitoneal injection), and after a right flank incision, the right renal hilum was clamped with a noninvasive microaneurysm clamp (Shanghai Medical Devices Co., Ltd., Shanghai, China) for 30 min. The left contralateral kidney was considered as a sham operation. The incision was temporarily closed during ischemia. After removal of the microaneurysm clip, the lateral abdominal incision was closed after visual confirmation of reperfusion. Body temperature was maintained using an adjustable heating pad. All mice were intraperitoneally injected with 0.5 mL of isotonic saline after surgery, and L-R, MOC, or MOC-R (250 μg/mL, 400 μL) were injected into different mice through the tail vein and the mice were executed at the indicated reperfusion time points.

### Histopathological assessment

Mice were executed at the indicated time points, and mouse kidneys were coronal sectioned, fixed in 10% buffered formalin, paraffin-embedded, and sectioned at a thickness of 3 μm sections were stained with hematoxylin and eosin to assess tissue damage. Kidney sections were blindly labeled and randomized for observation by two researchers. The extent of renal tubular injury was assessed semiquantitatively and pathologically on a scale of 0 to 4: 0 = no recognizable injury; 1 = single cell necrosis; 2 = necrosis of all cells in the adjacent convoluted tubule, with surrounding tubules surviving; 3 = necrosis confined to the distal one-third of the proximal convoluted tubule, with a band of necrosis spanning the endothelial layer; 4 = necrosis affecting all three segments of the proximal convoluted tubule.

### Immunofluorescence assay

Kidney frozen sections were processed according to standard protocols. Subsequently, the sections were incubated overnight with anti-DHE, KIM-1, NGAL, CD86/CD206, MPO, LC3 or TUNEL primary antibody provided by Beyotime Biotech. After that, the sections were rinsed three times with PBS and then incubated with secondary antibody for 1 h at room temperature. Cell nuclei were stained using DAPI. Finally, the slides were examined and imaged under a fluorescence microscope (Nikon80i, Tochigi, Japan).

### Western blotting

Tissues were rinsed with PBS twice and lysed in ice-cold RIPA buffer (Roche, Basel, Switzerland) containing phosphatase and protease inhibitors. Sample proteins were then subjected to 12% sodium dodecyl sulfate-polyacrylamide gel electrophoresis and transferred to nitrocellulose membranes. Membranes were probed with Beclin-1 and P62 antibodies from Beyotime Biotech. The relative quantity of proteins was determined by a densitometer software (ImageJ, NY).

### Masson staining

The kidney tissue was fixed in 4% paraformaldehyde. Slices of the paraffin-coated kidney samples were taken. Masson's Trichrome Stain Kit (Beyotime) was used on kidney tissues. Kidney sections were blindly labeled and randomized for observation by two researchers. The Masson staining was utilized to semi-quantitatively analyze the collagen volume fraction, which is defined as the proportion of collagen-positive blue area to the total tissue area. Image J software was employed for this purpose.

### Statistical analysis

Statistical significance was determined utilizing an ANOVA followed by Bonferroni's test correction using GraphPad Prism 10 (La Jolla, USA). The results are expressed as the mean ± standard deviation (SD). In every case, *p* < 0.05 was considered statistically significant.

## Supplementary Material

Supplementary figures.

## Abbreviations

AFM: atomic force microscopy
BUN: blood urea nitrogen
CAT: catalase
DAPI: 4',6-diamidino-2-phenylindole
DCFH-DA: 2',7'-dichlorodihydro-fluorescein diacetate
DLS: dynamic light scattering
DHE: dihydroethidium
DMPO: 5,5-dimethyl-1-pyrroline N-oxide
EPR: electron paramagnetic resonance
FBS: fetal bovine serum
FT-IR: flourier transform infrared spectroscopy
H/R: hypoxia/reoxygenation
H&E: hematoxylin and eosin
HK-2: Human kidney-2
I/R: ischemia-reperfusion
ICP-MS: inductively coupled plasma mass spectrometry
KIM-1: kidney injury molecule-1
MALDI-TOF: matrix assisted laser desorption/ionization time of flight
MOC: metal-organic cage
MOC-R: radical-functionalized metal-organic cage
MTT: methyl thiazolyl tetrazolium
NBT: nitrotetrazolium blue chloride
NGAL: neutrophil gelatinase-associated lipocalin
ROS: reactive oxygen species
SEC: size exclusion chromatography
SOD: superoxide dismutase
TEMPO: 2,2,6,6-tetramethylpiperidin-1-oxyl
WB: western blot
