# Supplementary material for: Ultrasmall radical metal organic cage as cascade antioxidant nanozyme for renal injury
Source: Theranostics. 2025 Jan 27;15(6):2564–78. doi: 10.7150/thno.105807 (PMC11840732; doi:10.7150/thno.105807)
Supplement: Supplementary file 1 — Supplementary figures. [file thnov15p2564s1.pdf]

Supplementary Material

Ultrasmall radical metal organic cage as cascade antioxidant nanozyme for renal injury

Cheng Huang[1†], Ziyu Liu[2†], Yucen Deng[1,3†], Xiaoyan Wang[4†], Qing Miao[1], Demei Sun[1], Xinyuan Zhu[1], Jinghui Yang[5*], and Youfu Wang[1*]

General information

All the reagents and solvents were commercially available and used as received.

NMR: The synthesized compounds were structurally confirmed by nuclear magnetic resonance (NMR) spectra in a Bruker Avance 500 (500 MHz) instrument using an internal deuterium lock for the residual protons in $CDCl_3$ at ambient probe temperature.

Mass spectra: The precise molecular weights of the synthesized compounds were recorded through mass spectra. Quadrupole Time of Flight LC/MS mass spectra were performed on Bruker impact II (Bruker, Germany). Matrix-assisted laser desorption ionization time of flight (MALDI-TOF) mass spectra were performed on a Bruker Speed MALDI-TOF 7090 (Bruker, Germany).

SEC: The molecular weights and polydispersity indexes (PDI) of ligands and MOCs were accessed by size exclusion chromatography (SEC) on a Malvern GPC using polymethylmethacrylate as standard. Tetrahydrofuran (THF) was used as the eluent at a flow rate of 1.0 mL/min at 35 °C.

FT-IR: Fourier transform infrared (FT-IR) spectra were recorded on a Bruker Tensor 27 FT-IR using ATR measurements for solids as neat samples.

DLS and Zeta Potential: The Particle Size and Zeta Potential Analyzer in a ZS90 instrument is mainly used to test particle size and size distribution and particle charges.

AFM: The morphologies and sizes of MOC-R were also estimated by atomic force microscopy (AFM) performed on MFP-3D (Oxford).

EPR: The Bruker A300 Electron Paramagnetic Resonance (EPR) instrument was used to detect the radical properties.

Synthetic details of L-R and MOC-R

The radical ligand, L-R, was synthesized according to **Scheme S1**.

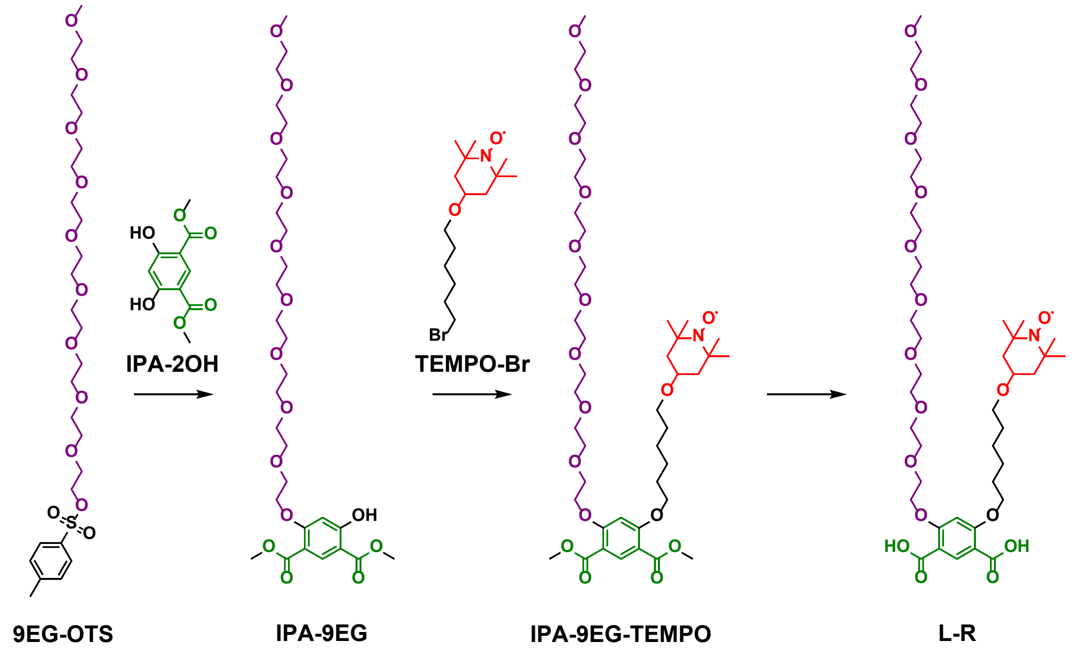

Scheme S1. The synthetic route of radical ligand, L-R.

9EG-OTS: 2,5,8,11,14,17,20,23,26-Nonaoxaoctacosan-28-ol (9EG) (2.50 g, 5.83 mmol, 1 eq) was placed in NaOH aqueous solution (5 mol/L, 17 mL) and cooled to 0 °C. Then *p*-toluenesulfonyl chloride (2.78 g, 14.58 mmol, 2.5 eq) was dissolved in 17 mL of THF and added dropwise to the above mixture. When the addition is completed, the reaction temperature returns to room temperature After reacting for 24 h at room temperature, the reaction solution was concentrated and then extracted three times using DCM (100 mL) and saturated saline water. The organic phase was collected, dried over $MgSO_4$, filtered, and concentrated by rotary evaporator. Then colorless oil **9EG-OTS** was obtained (95% yield). ^{1}H NMR (500 MHz, Chloroform-*d*, ppm) δ 7.78 (d, *J* = 8.0 Hz, 2H), 7.33 (d, *J* = 7.9 Hz, 2H), 4.14 (t, *J* = 4.8 Hz, 2H), 3.70 – 3.51 (m, 34H), 3.36 (s, 3H), 2.43 (s, 3H). MS(ESI): Calculated for $C_{26}H_{46}O_{12}S$, $[M+Na]^+$ 605.2602, found 605.2594.

IPA-9EG: Dimethyl 4,6-dihydroxyisophthalate (1.00 g, 4.42 mmol, 2 eq) was completely dissolved in 20 mL of DMF and then K_2CO_3 (1.53 g, 11.05 mmol, 5 eq) was added. The solution was heated to 100 °C for 10 min under N_2. **9EG-OTS** (1.29 g, 2.21 mmol, 1 eq) was dissolved in 50 mL of DMF under N_2 and slowly dripped into the above solution. After the addition, the reaction was stirred for 24 h. At the end of the reaction,

the reaction solution was concentrated and then extracted three times using DCM (100 mL) and saturated saline water to collect the organic phase. The organic phase was dried over $MgSO_4$, then filtrated and concentrated by rotary evaporator. Then, the oily **IPA-9EG** was obtained through purification by column chromatography on silica gel using DCM:MeOH (100:1, v/v) (48% yield). ^1H NMR (500 MHz, Chloroform-*d*, ppm) δ 8.41 (s, 1H), 6.48 (s, 1H), 4.20 (t, J = 4.9 Hz, 2H), 3.93 (d, J = 2.1 Hz, 3H), 3.84 (s, 3H), 3.77 (dd, J = 5.8, 3.7 Hz, 2H), 3.67 – 3.62 (m, 32H), 3.37 (s, 3H). MS(ESI): Calculated for $C_{29}H_{48}O_{15}$, $[M+Na]^+$ 659.2885, found 659.2888.

TEMPO-Br: NaH (0.42 g, 17.43 mmol, 3 eq) was placed in 4 mL of DMF under N_2 and stirred for 10 min. 2,2,6,6-Tetramethyl-4-hydroxy-1-piperidinyloxy radical (TEMPOL, 1.00 g, 5.81 mmol, 1 eq) was dissolved in 10 mL of DMF, added to above solution and stirred at room temperature for 3 h. 1,6-dibromohexane (4.25 g, 17.43 mmol, 3 eq) in 4 mL of DMF was dropped into the reaction solution at 0 °C. After the addition, the reaction solution was stirred at room temperature for 12 h. Then water was added to quench the excess NaH. The organic phase was then collected by extracting three times using DCM (60 mL) and water. The organic phase was dried over $MgSO_4$, filtrated, and concentrated by rotary evaporator. Finally, the oily **TEMPO-Br** was obtained through purification by column chromatography on silica gel using PE as eluent (52% yield). ^1H NMR (500 MHz, Chloroform-*d*, ppm) δ 3.59 – 3.52 (m, 1H), 3.45 – 3.38 (m, 4H), 1.97 – 1.89 (m, 2H), 1.89 – 1.82 (m, 2H), 1.59 – 1.55 (m, 2H), 1.49 – 1.43 (m, 4H), 1.40 – 1.33 (m, 2H), 1.22 (d, J = 2.0 Hz, 6H), 1.17 (s, 6H). MS(ESI): Calculated for $C_{15}H_{29}BrNO_2^{\bullet}$, [M] 334.1375, found 334.1376.

IPA-9EG-TEMPO: IPA-9EG (0.38 g, 0.6 mmol, 1 eq), **TEMPO-Br** (0.37 g, 1.1 mmol, 1.8 eq) and K_2CO_3 (0.29 g, 2.1 mmol, 3.5 eq) were placed in DMF (20 mL) under N_2, and stirred at 135 °C for 24 h. After cooling to room temperature, the solution was concentrated, filtrated and concentrated by rotary evaporator. Finally, the oily **IPA-9EG-TEMPO** was obtained through purification by column chromatography on silica gel using DCM:MeOH (80:1, v/v) as eluent (60% yield). ^1H NMR (500 MHz, Chloroform-*d*, ppm) δ 8.48 (s, 1H), 6.52 (s, 1H), 4.27 (t, J = 5.0 Hz, 2H), 4.09 (t, J = 6.5 Hz, 2H), 3.87 (d, J = 6.0 Hz, 6H), 3.82 – 3.79 (m, 2H), 3.72 – 3.63 (m, 32H), 3.57 (dd, J = 5.9, 3.4 Hz, 3H), 3.40 (s, 3H), 1.93 – 1.87 (m, 2H), 1.60 (dt, J = 28.0, 7.4 Hz, 4H), 1.50 – 1.44 (m, 2H), 1.40 (s, 6H), 1.31 (s, 6H), 1.28 (s, 2H). MS(ESI): Calculated for $C_{44}H_{76}NO_{17}^{\bullet}$, $[M+Na]^+$ 913.5005, found 913.5006

L-R: IPA-9EG-TEMPO (0.50 mg, 0.56 mmol, 1 eq) was added to LiOH aqueous solution (5 mol/L, 20 mL) and stirred at 70 °C for 3 h. After cooling down to room temperature, a diluted HCl solution (2 mol/L) was added to adjust the pH to 1. The solution was extracted with DCM for three times. The combined organic phase was dried over $MgSO_4$, filtered, concentrated to obtain **L-R** (91% yield). ^{1}H NMR (500 MHz, Chloroform-*d*, ppm) δ 8.53 (s, 1H), 6.50 (s, 1H), 4.39 – 4.30 (m, 2H), 4.10 (s, 2H), 3.93 (d, *J* = 5.1 Hz, 2H), 3.89 – 3.52 (m, 32H), 3.47 (t, *J* = 6.2 Hz, 2H), 3.38 (s, 3H), 1.85 (q, *J* = 7.8, 7.0 Hz, 2H), 1.61 (t, *J* = 6.9 Hz, 4H), 1.44 (d, *J* = 17.3 Hz, 12H), 1.27 (s, 2H). MS(ESI): Calculated for $C_{42}H_{72}NO_{17}^{\bullet}$, $[M+Na]^+$ 885.4692, found 885.4699.

MOC-R: L-R (0.10 g, 0.12 mmol, 1 eq) and $Cu(OAc)_2{\cdot}H_2O$ (0.024 g, 0.12 mmol, 1 eq) were dissolved in separated THF (5 mL). The two solutions were mixed together and stirred at room temperature for 3 h. The solution was concentrated and added dropwise into diethyl ether to precipitate the cage. The precipitation was centrifuged, washed, and dried to obtain **MOC-R** (86% yield).

L：IPA-9EG (0.50 mg, 0.79 mmol, 1 eq) was added to LiOH aqueous solution (5 mol/L, 25 mL) and stirred at 70 °C for 3 h. After cooling down to room temperature, the pH of the solution was adjusted to 1 using a dilute HCl solution (2 mol/L). The solution was extracted with DCM for three times. The combined organic phase was dried over $MgSO_4$, filtered, concentrated to obtain oil ligand, **L** (91% yield). MS(ESI): Calculated for $C_{27}H_{44}O_{15}$, $[M-H]^-$ 607.2607, found 607.2634.

MOC: L (0.10 g, 0.16 mmol, 1 eq) and $Cu(OAc)_2{\cdot}H_2O$ (0.032 g, 0.16 mmol, 1 eq) were dissolved in separated THF (8 mL). The two solutions were mixed and stirred at room temperature for 3 h. After concentration, the solution was added dropwise into diethyl ether to precipitate the cage. The precipitation was centrifuged, washed, and dried to obtain **MOC** (83% yield).

Supplementary figures

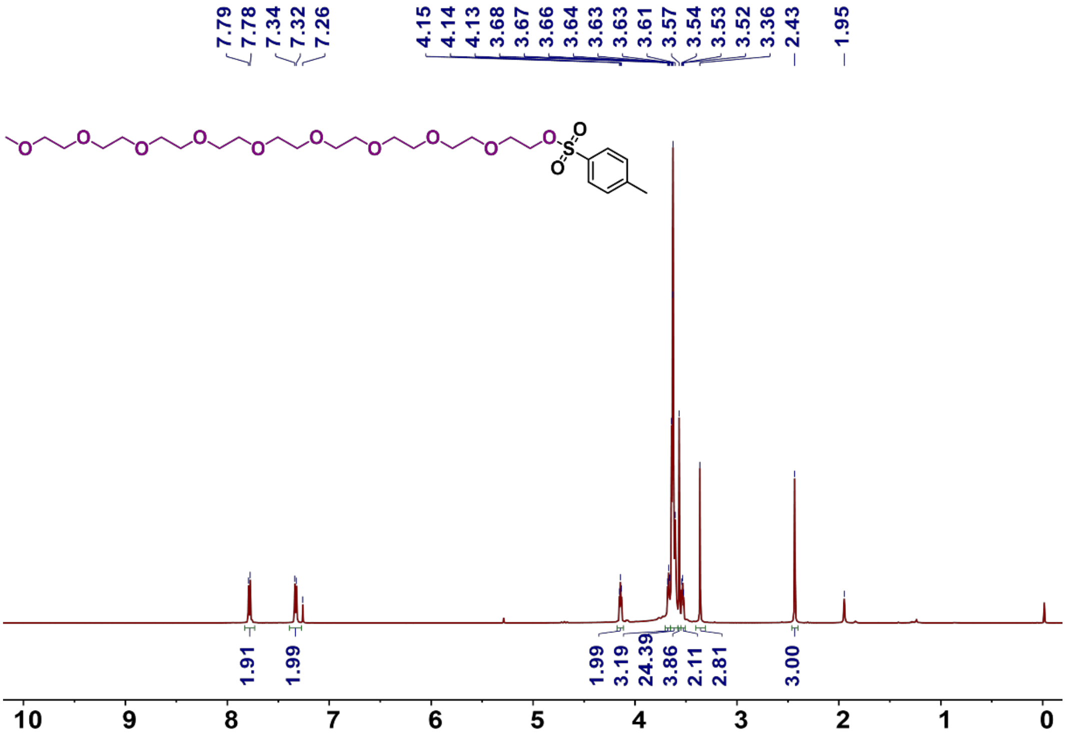

Figure S1. The ^{1}H-NMR spectra of **9EG-OTS** in $CDCl_3$.

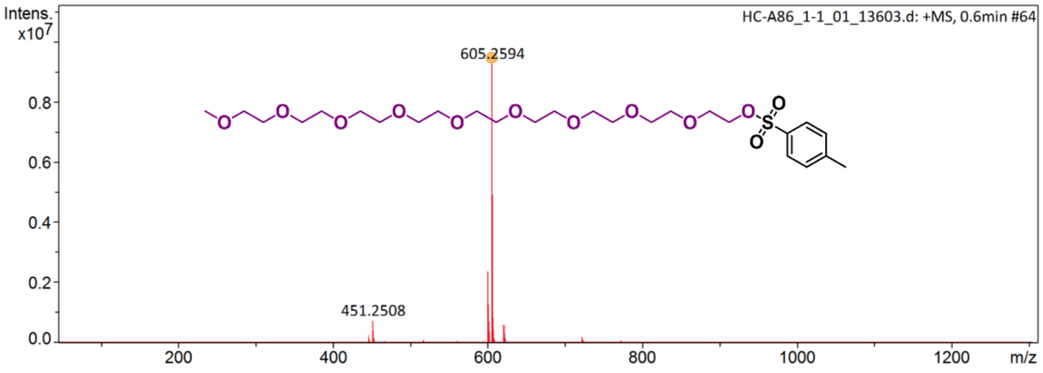

Meas. m/z	#	Ion Formula	m/z	Sum Formula	err [ppm]	mSigma	# mSigma	Score	rdb	e⁻ Conf	N-Rule
605.2594	1	C26H46NaO12S	605.2602	C26H46O12S	1.4	19.9	1	100.00	4.0	even	ok

Figure S2. The ESI mass spectra of **9EG-OTS**.

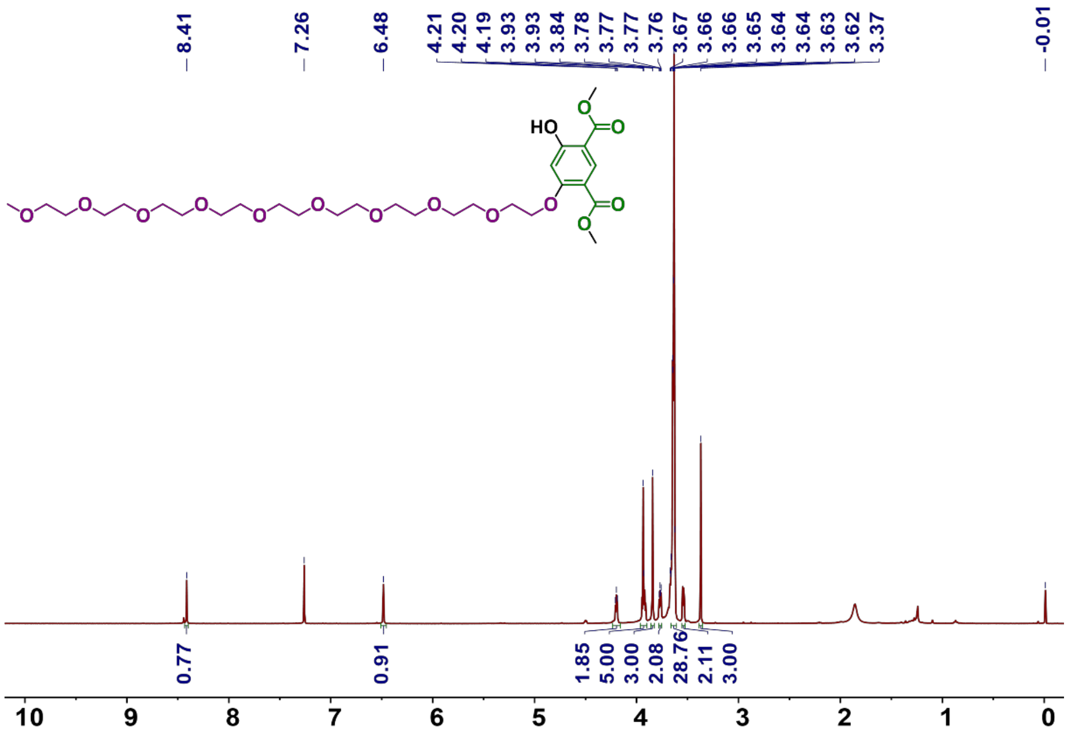

Figure S3. The ^{1}H-NMR spectra of **IPA-9EG** in $CDCl_3$.

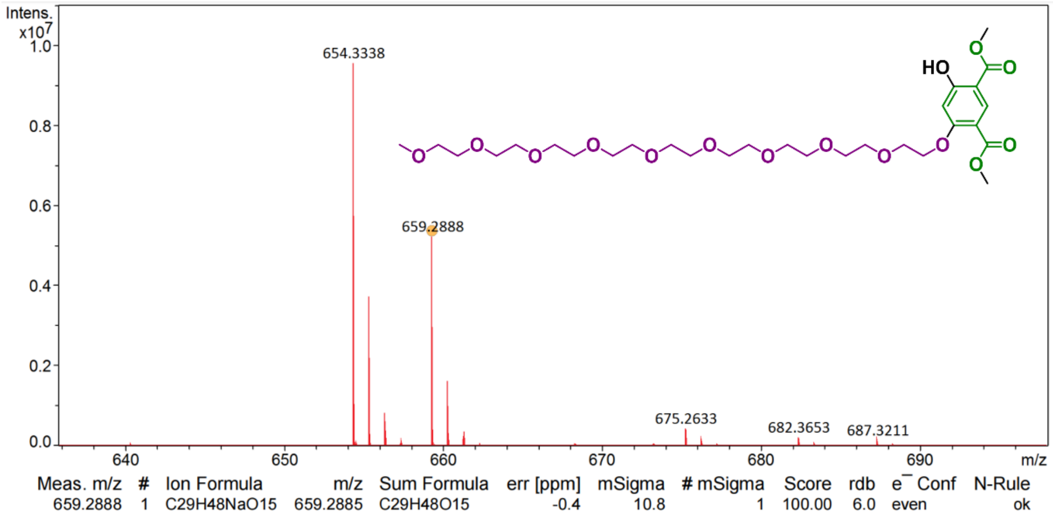

Figure S4. The ESI mass spectra of **IPA-9EG**.

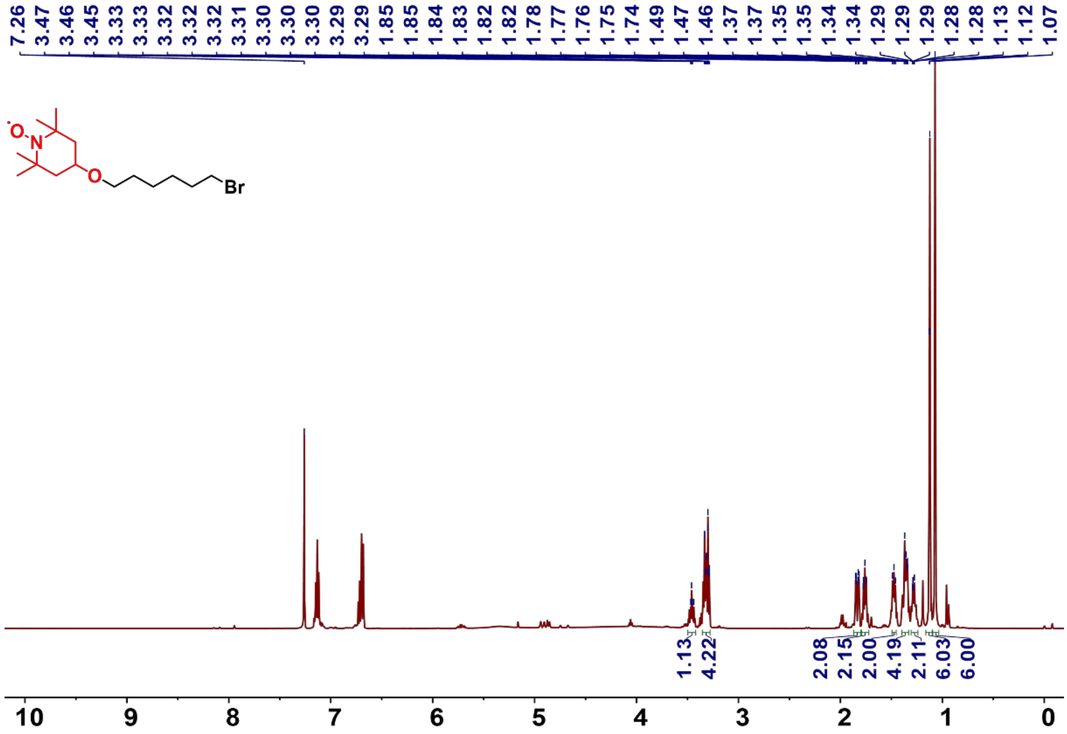

Figure S5. The ^{1}H-NMR spectra of **TEMPO-Br** in $CDCl_3$.

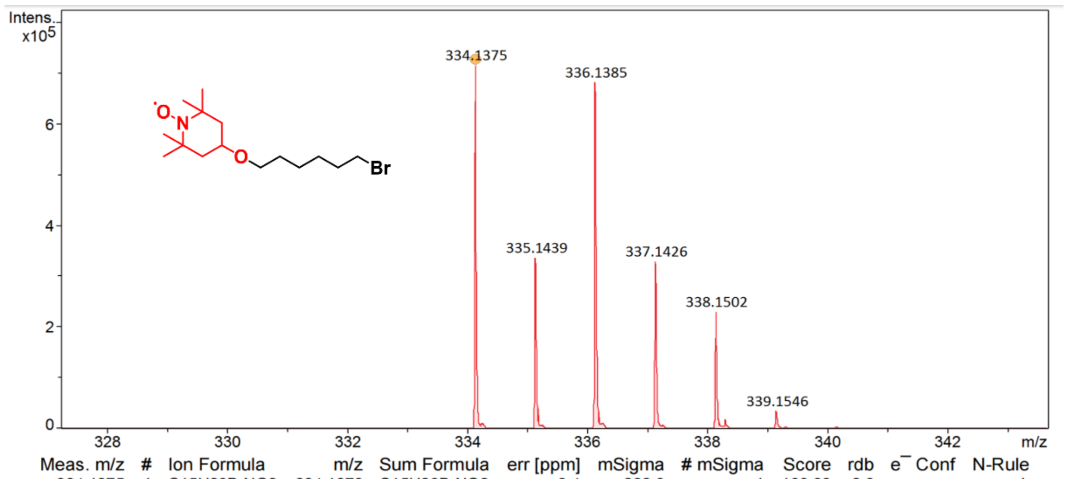

Meas. m/z	#	Ion Formula	m/z	Sum Formula	err [ppm]	mSigma	# mSigma	Score	rdb	e⁻ Conf	N-Rule
334.1375	1	C15H29BrNO2	334.1376	C15H29BrNO2	0.4	232.8	1	100.00	2.0	even	ok

Figure S6. The ESI mass spectra of **TEMPO-Br**.

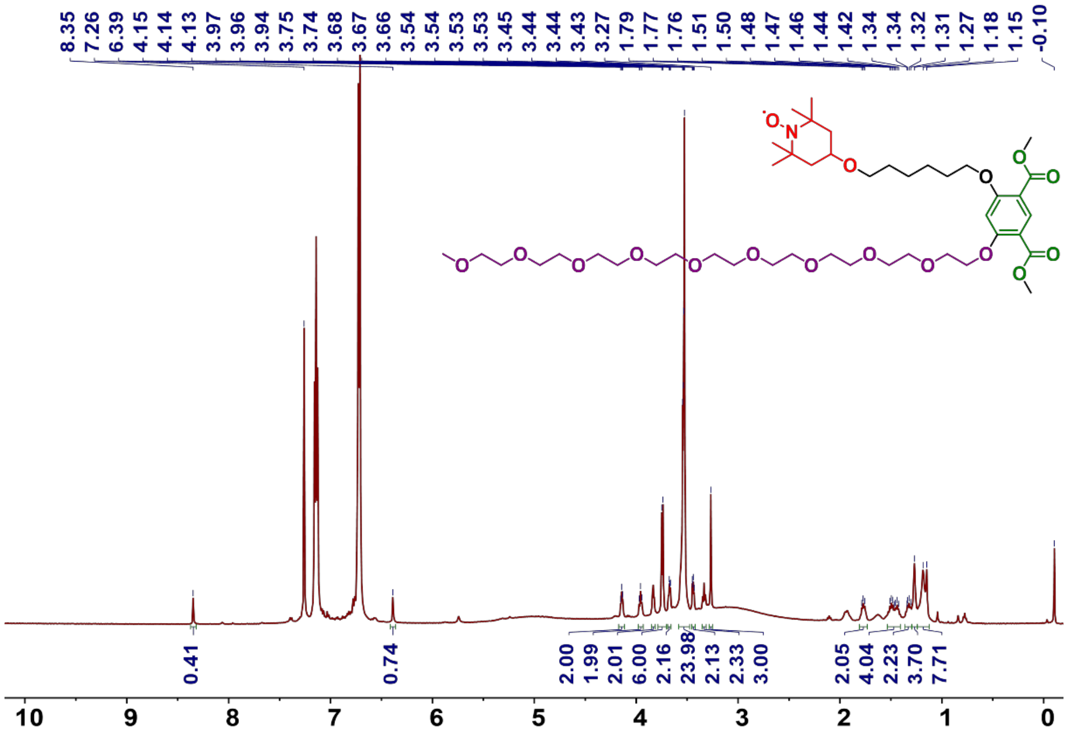

Figure S7. The ^{1}H-NMR spectra of **IPA-9EG-TEMPO** in $CDCl_3$.

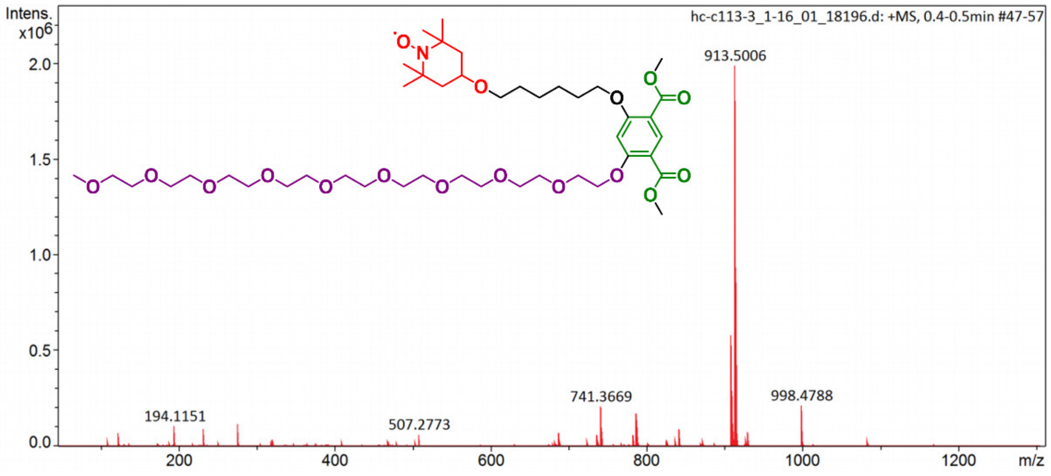

Figure S8. The ESI mass spectra of **IPA-9EG-TEMPO**.

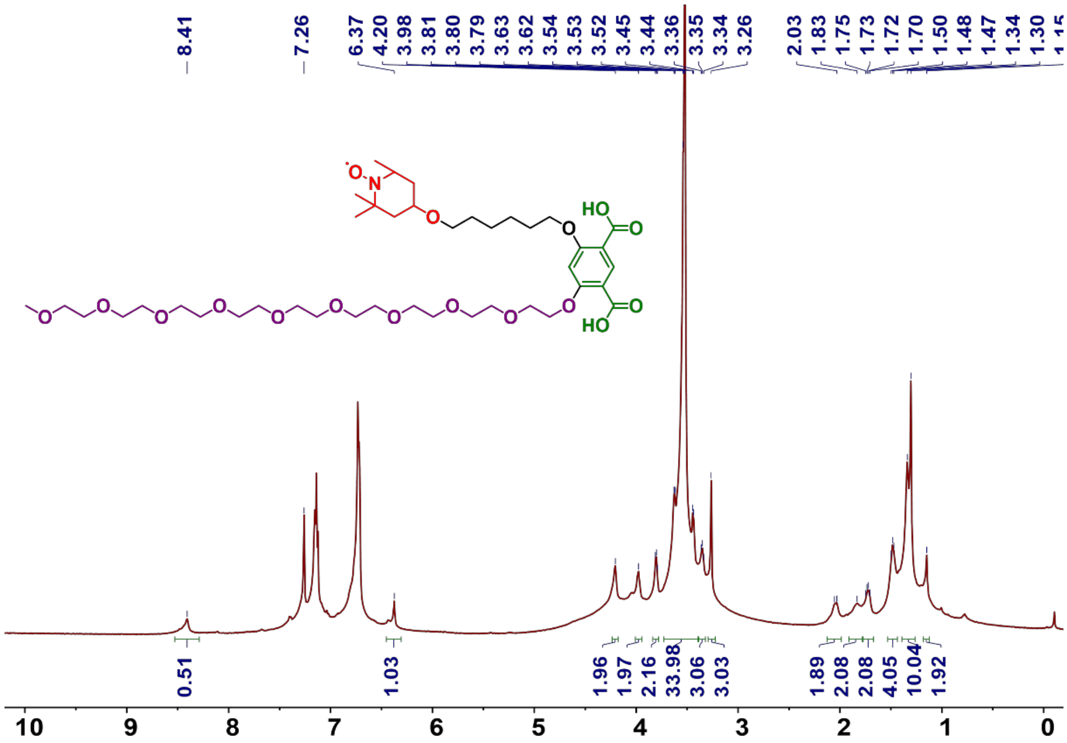

Figure S9. The ^{1}H-NMR spectra of **L-R** in $CDCl_3$.

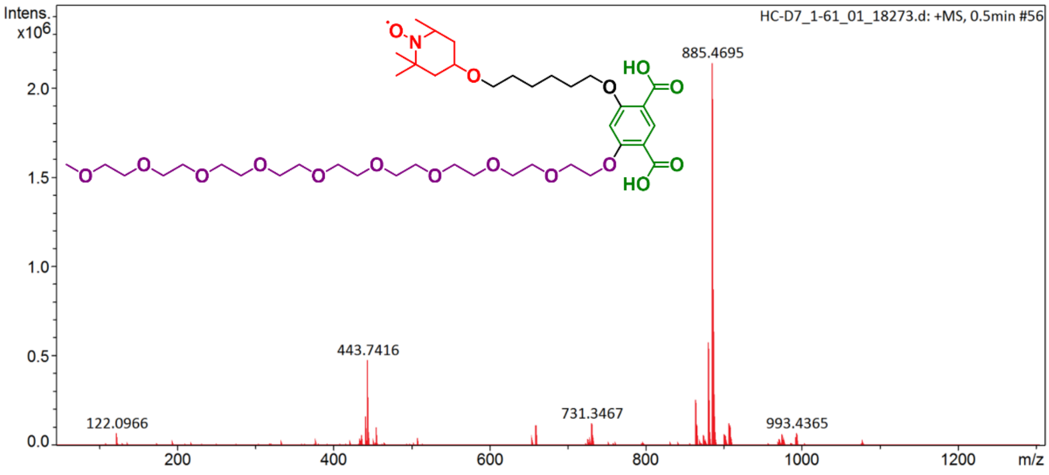

Figure S10. The ESI mass spectra of **L-R**.

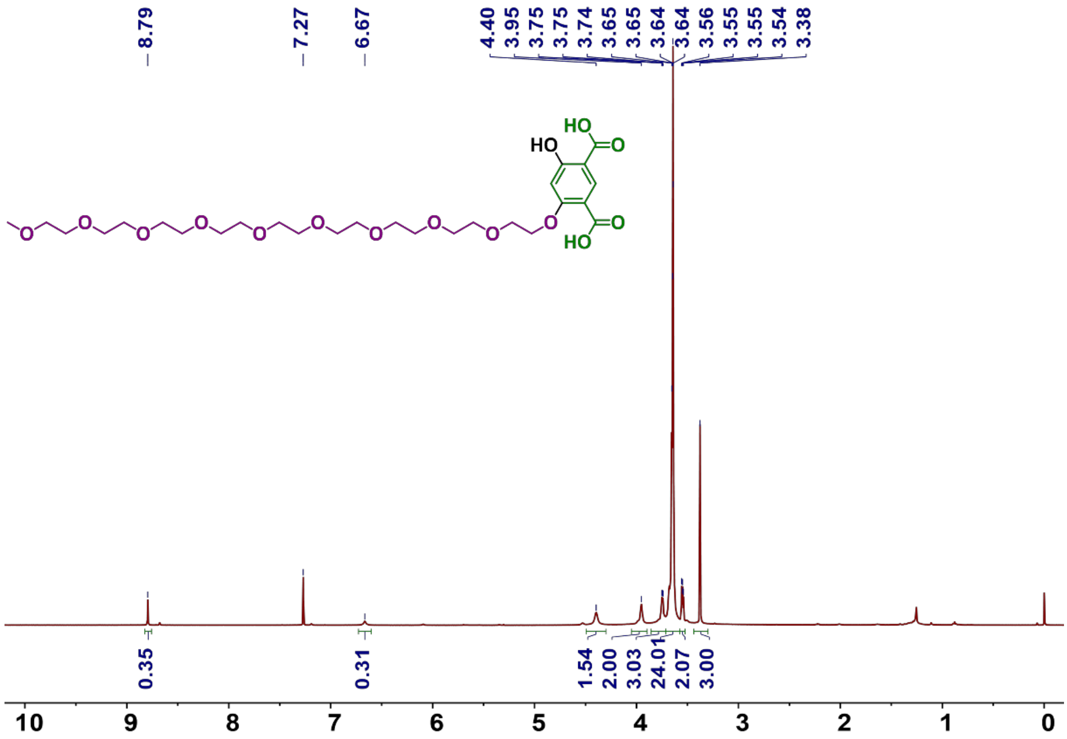

Figure S11. The ^{1}H-NMR spectra of **L** in $CDCl_3$.

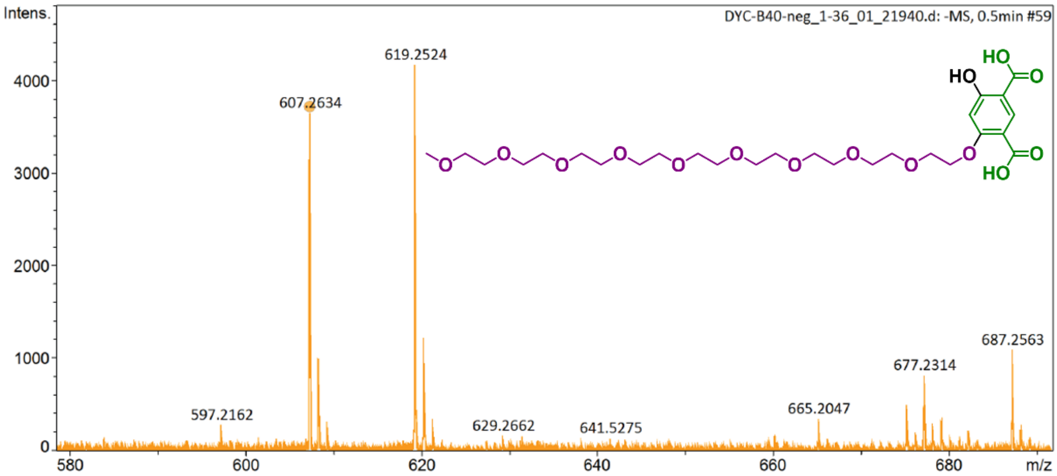

Meas. m/z	#	Ion Formula	m/z	Sum Formula	err [ppm]	mSigma	# mSigma	Score	rdb	e⁻ Conf	N-Rule
607.2634	1	C27H43O15	607.2607	C27H44O15	-4.4	16.9	1	100.00	6.0	even	ok

Figure S12. The ESI mass spectra of **L-R**.

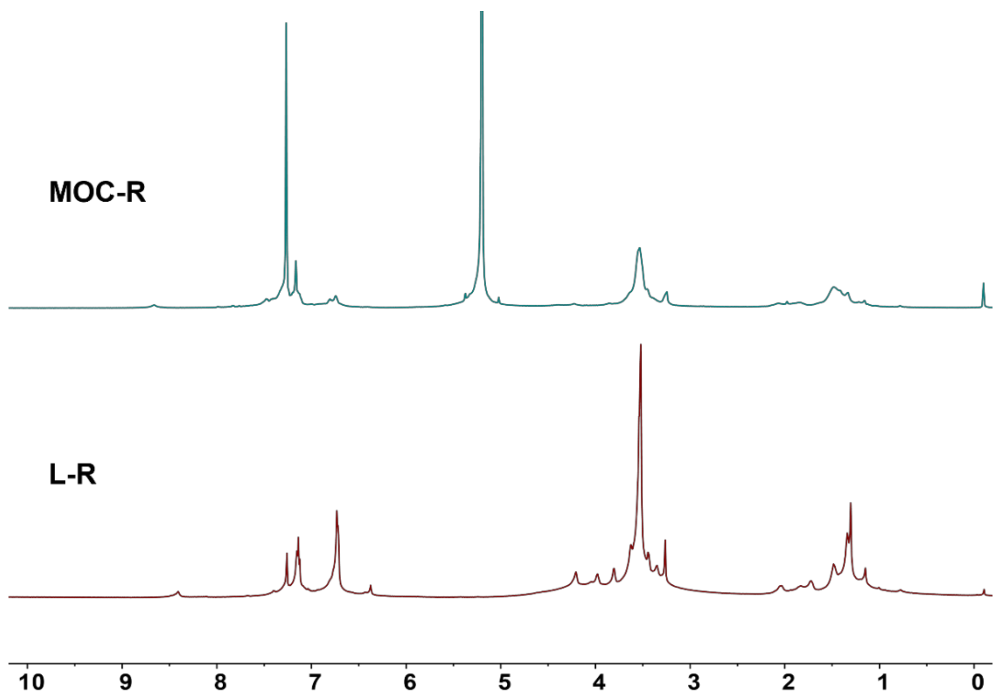

Figure S13. The ^{1}H-NMR spectra of **L-R** and **MOC-R** in $CDCl_3$.

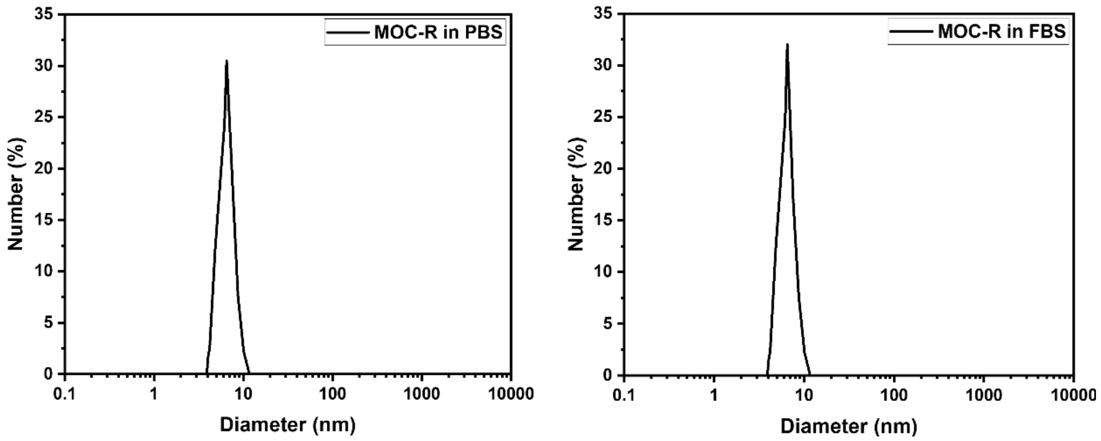

Figure S14. The DLS plots of MOC-R dissolved in PBS and FBS over a period of one day.

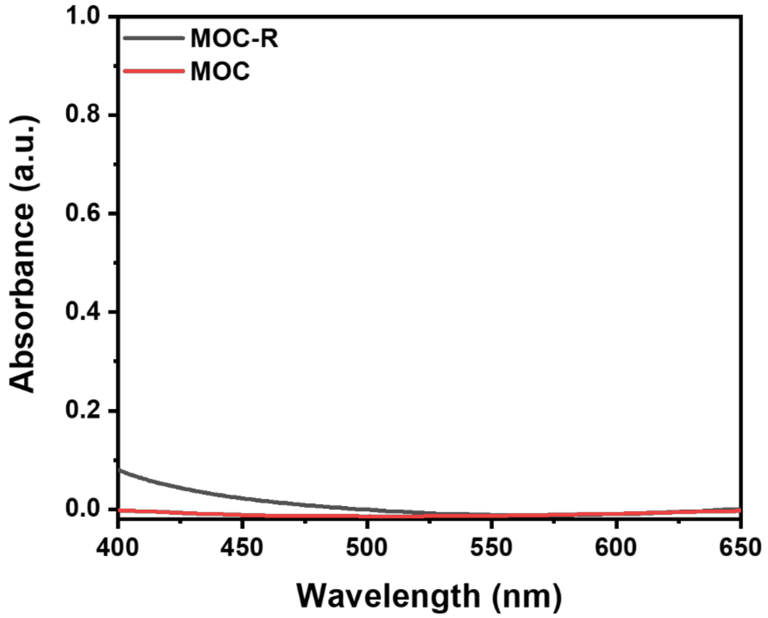

Figure S15. The UV-vis spectra of MOC and MOC-R in PBS.

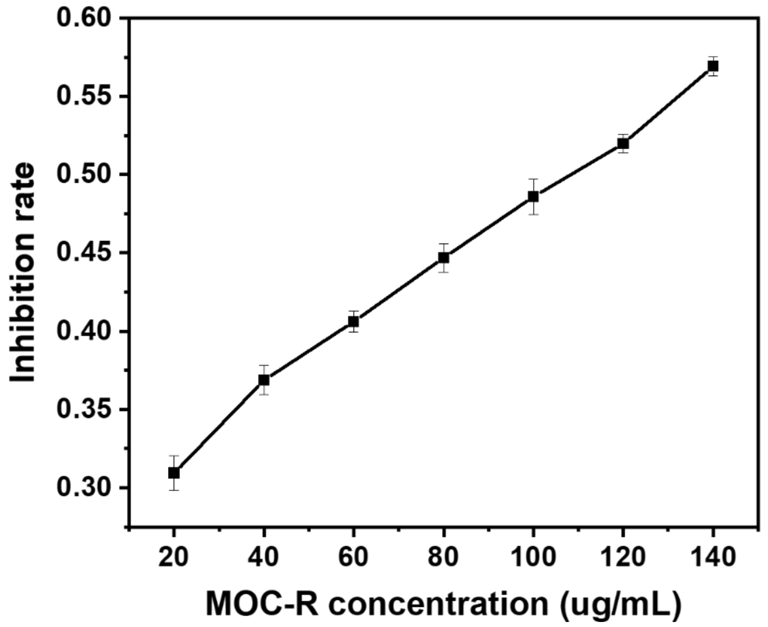

Figure S16. The SOD-like Inhibition rate of MOC-R toward $\bullet O_2^-$.

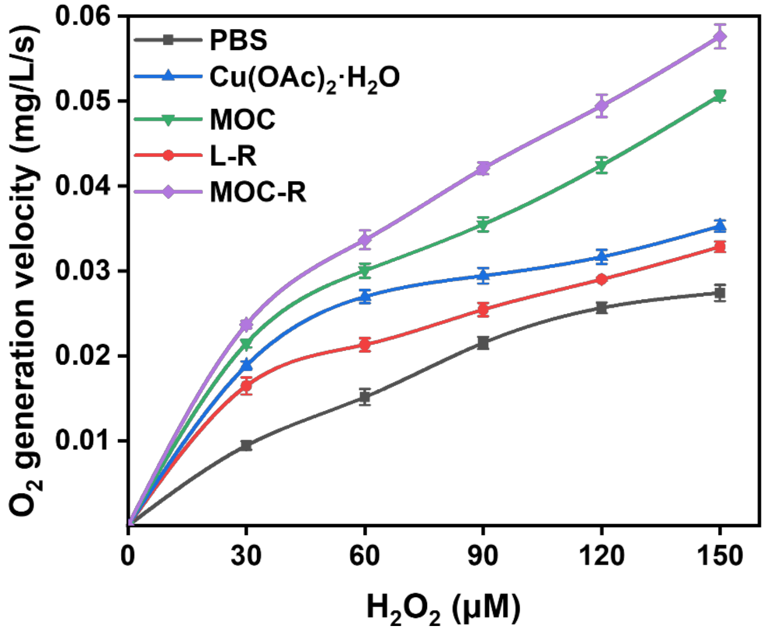

Figure S17. The H_2O_2 concentration dependent CAT-like activity of different groups.

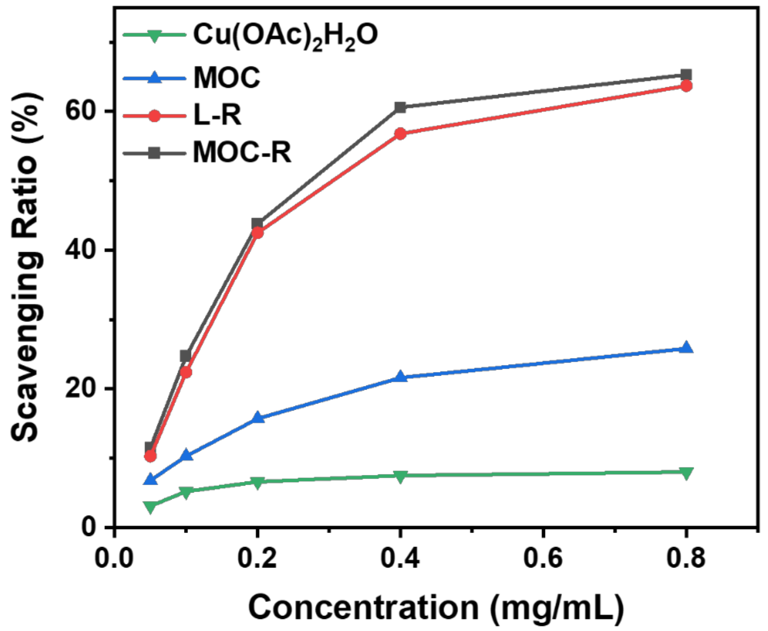

Figure S18. The hydroxyl radical scavenging capacity of different groups.

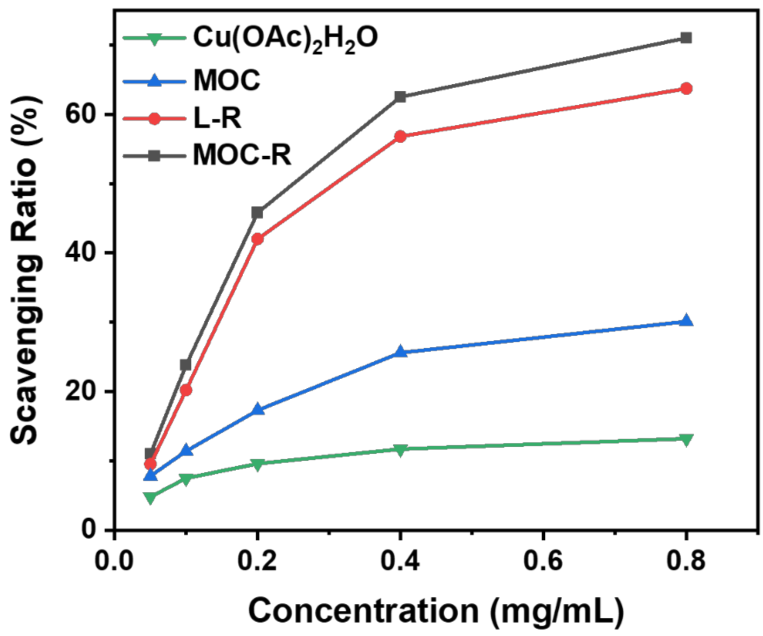

Figure S19. The ABTS radical scavenging capacity of different groups.

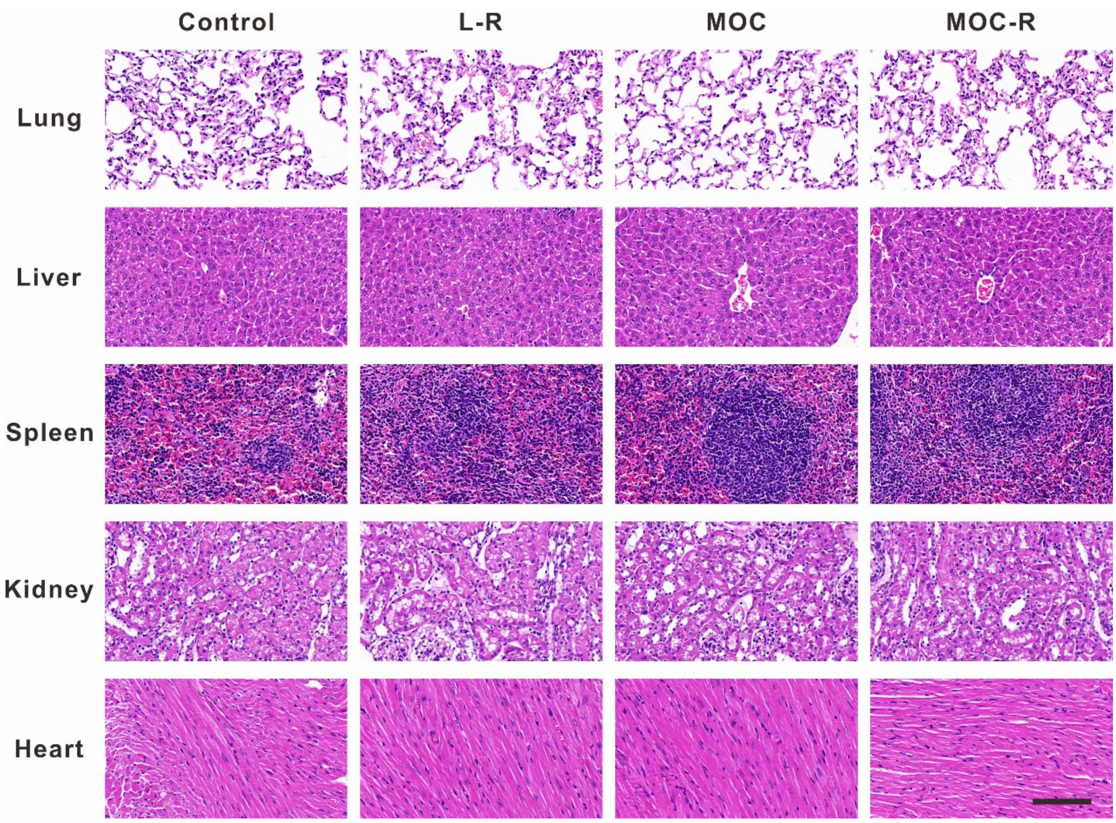

Figure S20. The evaluation of the biocompatibility of MOC-R (H&E staining of major organs). Scale bars represent 100 μm.

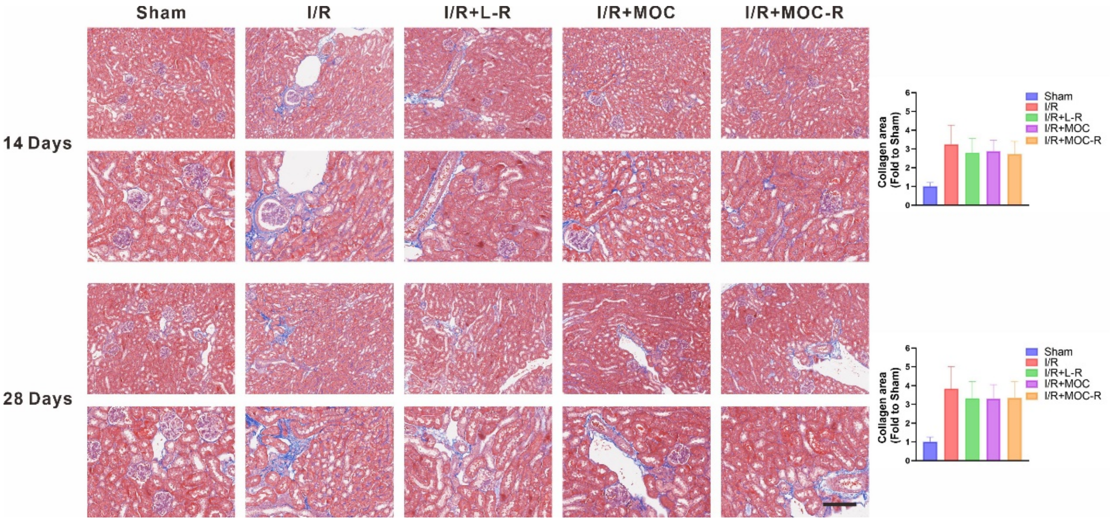

Figure S21. Representative Masson's trichrome staining of kidney sections at 14 and 28 days post-reperfusion (10× and 20×). Scale bars represent 100 μm.
